# Structures of synthetic nanobody–SARS-CoV-2–RBD complexes reveal distinct sites of interaction and recognition of variants

**DOI:** 10.21203/rs.3.rs-625642/v1

**Published:** 2021-06-16

**Authors:** Javeed Ahmad, Jiansheng Jiang, Lisa F. Boyd, Allison Zeher, Rick Huang, Di Xia, Kannan Natarajan, David H. Margulies

**Affiliations:** 1 Molecular Biology Section, Laboratory of Immune System Biology, National Institute of Allergy and Infectious Diseases, National Institutes of Health, Bethesda, MD, 20892, USA; 2 Laboratory of Cell Biology, Center for Cancer Research, National Cancer Institute, National Institutes of Health, Bethesda, MD, 20892, USA

**Keywords:** SARS-CoV-2, receptor-binding domain (RBD), X-ray crystal structures

## Abstract

The worldwide spread of severe acute respiratory syndrome coronavirus 2 (SARS-CoV-2) and emergence of new variants demands understanding the structural basis of the interaction of antibodies with the SARS-CoV-2 receptor-binding domain (RBD). Here we report five X-ray crystal structures of sybodies (synthetic nanobodies) including binary and ternary complexes of Sb16–RBD, Sb45–RBD, Sb14–RBD–Sb68, and Sb45–RBD–Sb68; and Sb16 unliganded. These reveal that Sb14, Sb16, and Sb45 bind the RBD at the ACE2 interface and that the Sb16 interaction is accompanied by a large CDR2 shift. In contrast, Sb68 interacts at the periphery of the interface. We also determined cryo-EM structures of Sb45 bound to spike (S). Superposition of the X-ray structures of sybodies onto the trimeric S protein cryo-EM map indicates some may bind both “up” and “down” configurations, but others may not. Sensitivity of sybody binding to several recently identified RBD mutants is consistent with these structures.

SARS-CoV-2, a β-coronavirus, is remarkable for its high infectivity, rapid world-wide dissemination, and evolution of highly infectious new variants ^1–4^. The virus exploits its trimeric spike (S) glycoprotein to adsorb to the host cell-surface receptor, angiotensin converting enzyme (ACE) 2 ^5^, resulting in proteolytic processing and conformational changes required for membrane fusion and cell entry ^6^. Understanding the fundamental molecular and cell biology and chemistry of the viral life cycle and the nature of the host immune response offer rational avenues for developing diagnostics, therapeutics, and vaccines ^7,8^. Emerging viral variants that exhibit increased infectivity and virulence emphasize the need for continued improvement in immunization and therapeutic approaches. Specifically, the B.1.1.7 (UK), B.1.351 (South Africa), P.1 (Brazil), and other strains demand careful attention ^9–14^. Exploring the detailed structures of anti-viral antibodies can provide critical understanding of means to attenuate viral adsorption and entry, and to prevent or retard ongoing infection and communal spread. An evolving database of X-ray and cryo-EM structures of the SARS-CoV-2 S and RBD and their interactions with ACE2 or various antibodies contributes to the design of effective antibodies or immunogens ^15^. Recent studies indicate the value of single domain antibodies derived from camelids (nanobodies) ^16^ or camelid-inspired synthetic libraries (sybodies) ^17,18^, and the potential effectiveness of multivalent constructs ^19^. Many properties of nanobodies make them well-suited for structural studies and drug development ^20^. Here, we take advantage of available sequences of five SARS-CoV-2 RBD-directed sybodies – Sb14, Sb15, Sb16, Sb45, and Sb68 (previously designated Sb#14, Sb#15, Sb#16, Sb#45, and Sb#68 ^18^). These sybodies effectively inhibit the ACE2–RBD interaction and neutralize viral infectivity ^18^. We describe binding studies and X-ray structures of binary complexes of Sb16–RBD and Sb45–RBD; ternary complexes of Sb14-RBD-Sb68 and Sb45– RBD–Sb68; and Sb16 unliganded. In addition, we report cryo-EM structures of Sb45 complexed with trimeric S and evaluate sybody interactions with several mutant RBD representative of newly evolving variants.

## Results

### Binding and affinity analysis.

**Fig. 1|:**
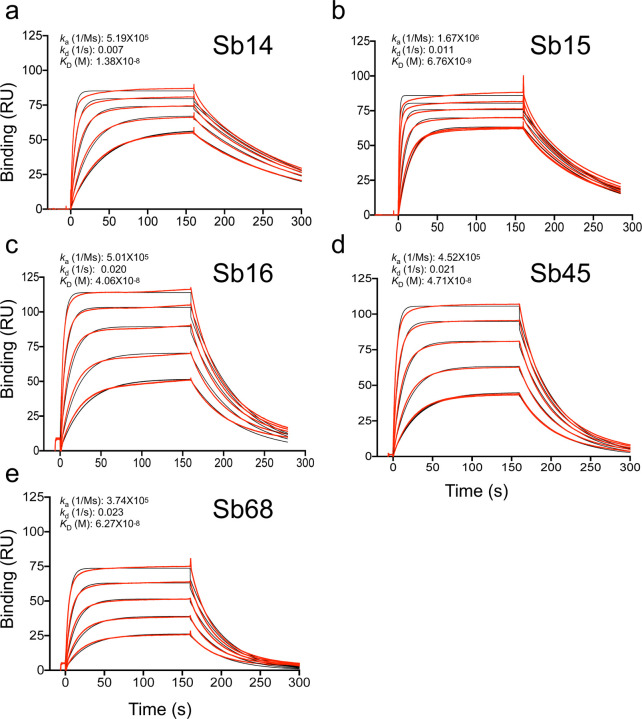
Sybodies bind RBD with *K*_D_ values in the nanomolar range. RBD was coupled to a biosensor chip as described in Methods. Graded concentrations (31 to 500 nM) of each of the indicated sybodies were offered to the coupled surface (from t=0 to t=160 s), followed by buffer washout, and measurement of net binding (in resonance units, RU). Experimental curves were fit by global analysis using BIAeval 2.0 (Cytiva). Curves shown are representative of at least two determinations.

Sybodies were expressed in *E. coli* and purified via metal-affinity chromatography to high purity. These sybodies behaved as monomers by size exclusion chromatography (SEC) ^21^ (Extended Data Fig. 1), and we confirmed their activity in binding to the bacteria-expressed RBD as visualized by SEC (Extended Data Fig. 1). As determined by surface plasmon resonance (SPR), all five sybodies bind to immobilized RBD with *K*_D_ values of 6.8 to 62.7 nM, consistent with previous determinations using RBD-YFP or RBD-Fc molecules by related techniques ^18^ (Fig.1).

### Structure of Sybody-RBD binary and ternary complexes.

To gain insight into the precise topology of the interaction of four of these sybodies with the RBD, we determined crystal structures of their complexes: Sb16–RBD, Sb45–RBD; the ternary Sb45–RBD–Sb68 and Sb14– RBD–Sb68; and of Sb16 alone. These crystals diffracted X-rays to resolutions from 1.7 to 2.6 Å (Table 1). After molecular replacement, model building, and crystallographic refinement (see Methods), we obtained structural models that satisfied standard criteria for fitting and geometry (Table 1). Illustrations of the quality of the final models as compared with the electron density maps are shown in Extended Data Fig. 2.

**Fig. 2|:**
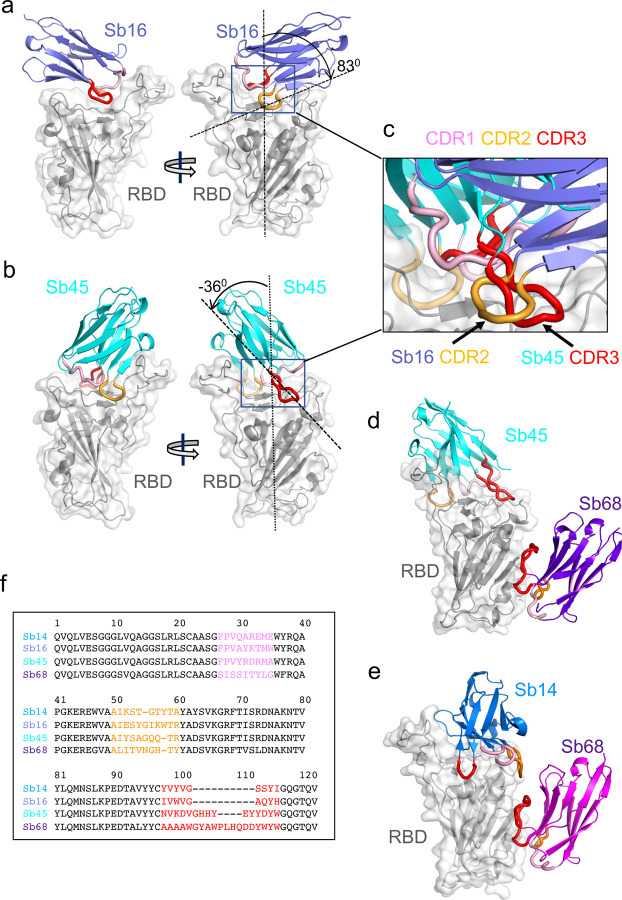
Overall structures of Sb14, Sb16, Sb45 and Sb68 complexed with SARS-CoV-2 RBD. Ribbons (sybodies) and ribbons plus surface (RBD) representations of the complex of (**a**) Sb16 (slate) with RBD (grey) (7KGK); (b) Sb45 (cyan) with RBD (7KGJ), (d) Sb45 and Sb68 (purple) with RBD (7KLW) and (e) Sb14 (blue) and Sb68 (magenta) with RBD (7MFU). Sb16-RBD and Sb45-RBD superimposed based on the RBD are shown in (c) to highlight CDR loops, which are color coded as CDR1 (pink), CDR2 (orange) and CDR3 (red). The CDR2 of Sb16 and CDR3 of Sb45 interact similarly with the RBD surface. Panel (f) shows a sequence alignment of the four sybodies.

**Fig. 3|:**
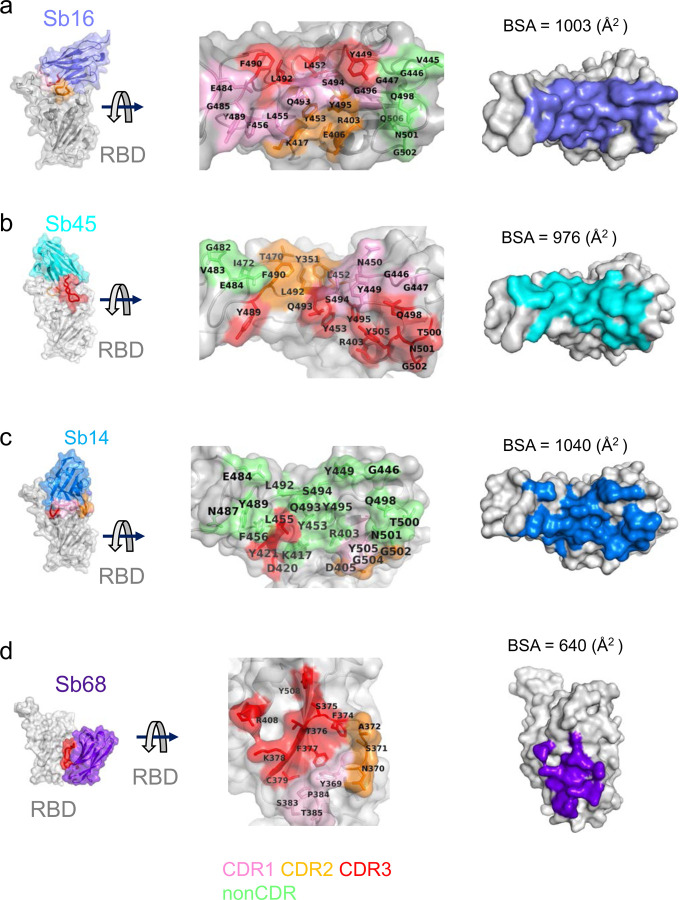
Interfaces and interactions of sybodies with RBD. (**a**) Sb16-RBD, (b) Sb45-RBD, (c) Sb14-RBD, and (d) Sb68-RBD. (Individual contacting residues are listed in Supplementary Table 1). CDR1, CDR2, CDR3 regions are painted pink, orange and red respectively. Additional non-CDR region contacting residues are colored lime. On the RBD surface, the epitopic residues that contact the sybodies are colored according to the sybody CDR.

The structure of the RBD of these complexes (Fig. 2a, b) revealed little difference between insect-expressed ^22^ and our bacteria-expressed and refolded RBD. Each of the sybodies has a barrel of two β-sheets stabilized by a single disulfide-linked loop of 75 or 76 amino acids characteristic of an IgV fold ^23,24^. The Sb16–RBD complex (Fig. 2a, 3a) illustrates that CDR2 (residues 50–60) and CDR3 (residues 98–106) bestride the saddle-like region of the ACE2-binding surface of the RBD (see sequence alignment in Fig. 2f). Sb16 angulates over the RBD by 83°. However, Sb45 (Fig. 2b and 3b) straddles the RBD saddle in the opposite orientation, at an angle of −36°, and frames the interface with CDR2 (residues 50–59) and CDR3 (residues 97–111). CDR1s of both sybodies (residues 27–35) lie between the CDR2 and CDR3 loops. Superposition of the two structures, based on the RBD, emphasizes the diametrically opposite orientation of the two (Fig. 2c), revealing that the CDR2 of Sb16 and CDR3 of Sb45 recognize the same epitopic regions.

Exploring conditions using mixtures of two or three sybodies and the RBD, we obtained crystals and solved the structures of ternary complexes consisting of Sb45–RBD–Sb68 at 2.6 Å resolution (Table 1 and Fig. 2d) and Sb14–RBD–Sb68 at 1.7 Å resolution (Fig. 2e). The refined models revealed that while Sb14 and Sb45 interact with the ACE2 interface of the RBD, Sb68 binds the RBD at a distinct site (Fig. 2d, e). In the ternary complex, Sb45 binds in an identical orientation to that observed in the binary Sb45–RBD structure (RMSD of superposition, 0.491 Å for 1981 atoms), but Sb68 addresses a completely different face of the RBD – similar to that bound by Fab of CR3022 on RBD of SARS-CoV-2 ^25^ and by V_HH_72 on RBD of SARS-CoV-1 ^26^. Of particular interest, whereas Sb45 CDR2 and CDR3 span the RBD saddle as noted above, the distinct contacts of Sb68 to the RBD are through the longer CDR3, with only minor contributions from CDR1 and CDR2. Walter et al visualized similar distinct interactions in cryo-EM maps of two sybodies (Sb15 and Sb68) bound to S protein with local resolution of 6–7 Å ^18^. Similarly, Sb14, which interacts via distinct sybody residues with the RBD at the ACE2 site (see description below), still permits Sb68 to bind to its epitope as seen in the Sb45–RBD–Sb68 structure (Fig. 2d).

Scrutiny of the different interfaces provides insights into the distinct ways each sybody exploits its unique CDR residues for interaction with epitopic residues of the RBD (Fig. 3). (Compilation of the contacting residues for each of the four sybodies to the RBD is provided in Supplementary Table 1). Both Sb16 and Sb45 use longer CDR2 and CDR3 to straddle the RBD, positioning CDR1 residues over the central crest of the saddle (Fig. 2a–c; Fig.3a,b and Supplementary Table 1). Also, several non-CDR residues (Y37, E44, and W47 for Sb16), derived from framework 2 ^27^, provide additional contacts to the RBD (see Supplementary Table 1). By contrast with Sb16 and Sb45, Sb14, despite interacting with a large surface area of the RBD, uses both CDR2 and CDR3 on the same side and exploits many non-CDR residues, particularly sheets of β-strand as its binding surface (Fig. 3c and Supplementary Table 1). The interface of Sb68 with RBD (Fig. 3d) is quite different, predominantly exploiting nine CDR3, four CDR2, and one CDR1 residues at the interface (see Supplementary Table 1).

### Sybodies block ACE2-RBD interaction in discrete ways.

**Fig. 4|:**
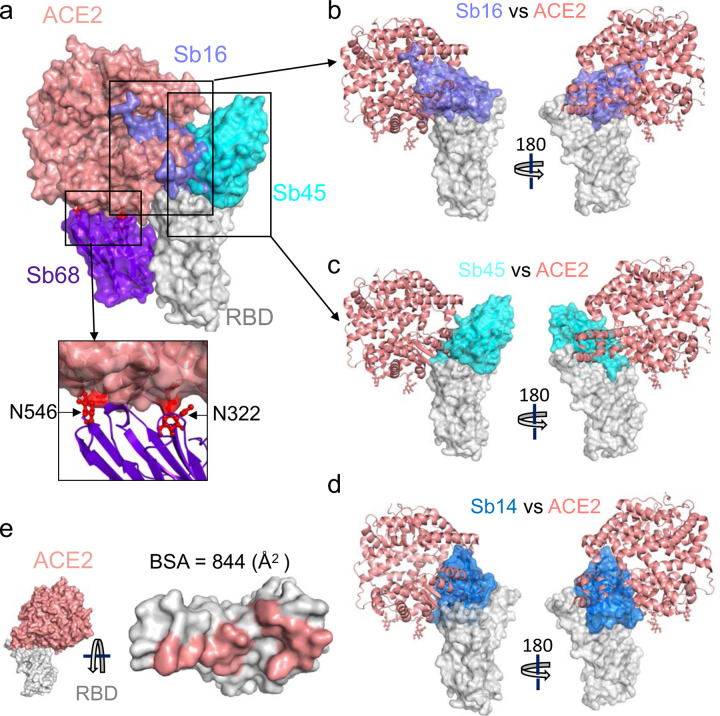
Sybodies clash with ACE2 in RBD complex structures. (a) Sb16 (slate), Sb45 (cyan), Sb14 (blue), and Sb68 (purple) – RBD complexes were superposed on the ACE2–RBD structure (salmon) (6M0J) based on the RBD. Views of Sb16 (b), Sb45 (c), and Sb14 (d) are shown alone as well. Sb14 and Sb16 are buried inside ACE2; Sb45 is partially buried in ACE2; and Sb68 has major clashes with two N-glycan sites (N322 and N546) of ACE2 (inset). (e) Epitopic area (on RBD) captured by ACE2 (salmon) is indicated along with its BSA.

To evaluate the structural basis for the ability of these four sybodies to block the interaction of RBD with ACE2, we superposed each of three sybody–RBD structures onto the ACE2–RBD structure and examined the steric clashes (Fig. 4a). Sb16 and Sb45 directly impinge on the ACE2 binding site, offering a structural rationale for their viral neutralization capacity ^18^. Sb68, which also blocks viral infectivity, binds to RBD at a site which appears to be noncompetitive for ACE2 binding. The carbohydrate at ACE2 residues N322 and N546 provides an explanation (Fig. 4a).

To compare the epitopic areas captured by these sybodies, we evaluated the buried surface area (BSA) interfaces between RBD and ACE2 or the sybodies. The BSA at the ACE2–RBD, Sb14–RBD, Sb16–RBD, Sb45–RBD, and Sb68–RBD interfaces are 844 Å^2^, 1,040 Å^2^, 1,003 Å^2^, 976 Å^2^, and 640 Å^2^, respectively (Fig. 3a–e). Sb16 and Sb45 capture more surface area than ACE2 or other published nanobody or sybody–RBD complexes (see Supplementary Table 2). The interface with Sb68 is the smallest (640 Å^2^) (Fig. 3d). The total BSA captured by Sb45 and Sb68 in the ternary complex is 1,650 (1,010 plus 640) Å^2^ (Supplementary Table 2) and is consistent with the view that a linked bispecific sybody, as described by Walter et al ^18^, would exert strong avidity effects. Supplementary Table 2 summarizes these BSA values and those of other nanobody–RBD interactions.

Although Sb68 reveals the smallest BSA with the RBD and binds at a distinct site, it still blocks ACE2 binding. A reasonable explanation for the ability of Sb68 to block the ACE2–RBD interaction arises on inspection of the sites where Sb68, bound to the RBD, might clash with ACE2. Scrutiny of a superposition of Sb68–RBD with ACE2–RBD reveals several areas of steric interference. Sb68 loop 40–44 clashes with amino acid side chains of ACE2 (residues 318–320 and 548–552), loop 61–64 with ACE2 N322 carbohydrate, and loop 87–89 (a 3,10 helix) with ACE2 N546 carbohydrate as well as residues 313 and 316–218 (Fig. 4a). The ACE2 used in the crystallographic visualization of ACE2–RBD ^28^ was expressed in *Trichoplusia ni* insect cells, which produce biantennary N-glycans terminating with N-acetylglucosamine residues ^29,30^. Electron density was observed only for the proximal N-glycans at residues N322 and N546, but larger, complex, non-sialylated, biantennary carbohydrates have been detected in glycoproteomic analysis of ACE2 in mammalian cells ^31^. These carbohydrates are highly flexible, adding greater than 1500 Da at each position, and are larger than the single carbohydrate residues visualized in the crystal structure. Additionally, molecular dynamics simulations of ACE2–RBD implicated the direct interaction of carbohydrate with the RBD ^32^. Thus, the ability of Sb68 to impinge on ACE2 interaction with RBD likely involves the steric clash of the N322- and N546-linked glycans.

We also obtained a 1.9 Å structure of free Sb16 (Extended Data Fig. 3). Remarkably, the CDR2 of Sb16 shows Y54 in starkly different positions in the unliganded structure as compared to the complex: the Cα carbon is displaced by 6.0 Å, while the Oη oxygen of Y54 is 15.2 Å distant, indicative of dynamic flexibility.

### Analysis of cryo-EM maps of Sb45-trimeric S complexes.

**Fig. 5|:**
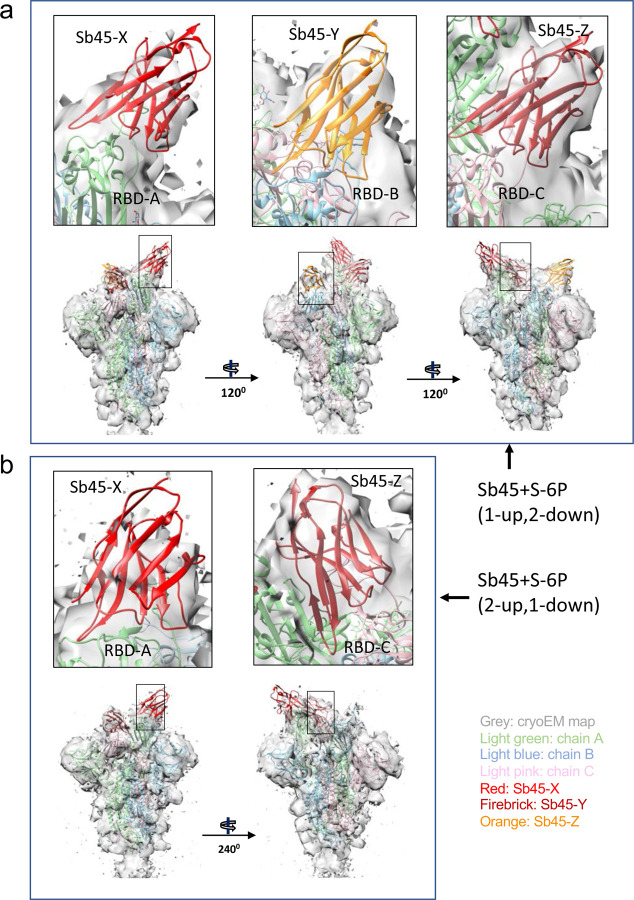
X-ray model of sybody superposed on cryo-EM Structures of SB45–S-6P. (a) Model of Sb45+S-6P (1-up, 2-down) is fitted to the map with Sb45-X bound to RBD-A (up), Sb45-Y to RBD-B (down), and Sb45-Z to RBD-C (down), and CC (Sb45-X/Sb45-Y/Sb45-Z) are 0.52/0.49/0.57 respectively; (b) Model of Sb45+S-6P (2-up, 1-down) is fitted to the map with Sb45-X bound to RBD-A (up), and Sb45-Z bound to RBD-C (down), and CC (Sb45-X/Sb45-Z) are 0.47/0.70 respectively.

To gain further insight into the interaction of Sb45 with the full S protein, we prepared complexes of Sb45 with HexaPro S (S-6P) ^33^ and acquired cryo-EM images as described in Methods. All image processing, 2D class, 3D reconstruction, and map refinements were performed with cryoSPARC ^34–37^, model fitting with Chimera ^38^ and refinement with PHENIX ^39^. We identified two conformations of S-6P with RBD in either a 1-up, 2-down (7N0G/EMD-24105) or 2-up, 1-down (7N0H/EMD-24106) position as determined by 3D classification (3D Ab-initio reconstruction) (Extended Data Fig.4). We have built in additional loops of the NTD and glycans based on the models of 6XKL, 7KGJ, and 7B62. We used unsharpened maps for the model refinement. The overall correlation coefficients (CC) (mask/volume/peaks) of models for 7N0G and 7N0H are 0.84/0.84/0.77 and 0.83/0.83/0.77 respectively. The model quality is shown in Table 2. There are three Sb45s binding to the 1-up, 2-down form of S-6P (7N0G/EMD-24105); one binds the up position of RBD, two bind the down position of RBD (Fig. 5a) with CC values of 0.51, 0.49 and 0.58 respectively (Extended Data Fig. 5a–c). Only two Sb45s bind to the 2-up, 1-down form of S-6P (7N0H/EMD-24106), with one on the up position of the RBD, and the other on the down position of the RBD (Fig. 5b) with CC values of 0.51 and 0.71 respectively (Extended Data Fig. 5d,e). It seems that Sb45 can bind all the down positions of the RBD. In particular, Sb45-Z binds well to RBD-C with higher CC values (Extended Data Fig. 5c,e), with additional contacts to the neighboring (up position) RBD-A (Fig. 5a). These variations in saturation of the available conformations by Sb45 reflect the mobility of the RBD. Notably, the interfaces between Sb45 and RBD of S-6P are the same as those in the crystal structure (7KGJ) (Fig. 2). Moreover, the RBD domains are compressed down towards the center of S, approximately 2–4 Å as compared with uncomplexed S-6P (6XKL).

### Superposition of sybodies on trimeric spike protein models.

To gain additional insight into the structural consequences of the interactions of each of these sybodies with a trimeric S protein, we superposed each of the individual sybody–RBD complexes on S-6P of our cryo-EM structures (7N0G and 7N0H) (see Extended Data Fig. 6). Sb16 and Sb45 may dock on all three RBDs in the trimeric S in any of the four configurations, without any apparent clash (Extended Data Fig. 6a,b). Sb14, however, reveals clashes when the Sb14–RBD complex is superposed on trimeric S in any down position (Extended Data Fig. 6e). Sb68 could not be superposed without clashes to any RBD of the 3-down or to the 1-up, 2-down position. The only permissible superpositions were to two in the 2-up, 1-down; and to all three in the 3-up position (Extended Data Fig. 6f). For paired sybodies, Sb16 and Sb68 (Extended Data Fig. 6b), or Sb45 and Sb68 (Extended Data Fig. 6d), superposition was possible without clashes, with two or more RBDs in the up conformation. Walter et al ^18^ suggested that a covalent bispecific Sb15–Sb68 reagent could bind S in both the 2-up and 3-up configurations, based on cryo-EM maps of complexes of S with Sb15 and Sb68. It appears that Sb16 binds to S in an orientation similar to but in detail distinct from that of Sb15. This analysis demonstrates an advantage of the small size of sybodies or nanobodies in accessing epitopic regions of S.

### Binding to RBD mutants.

**Fig. 6|:**
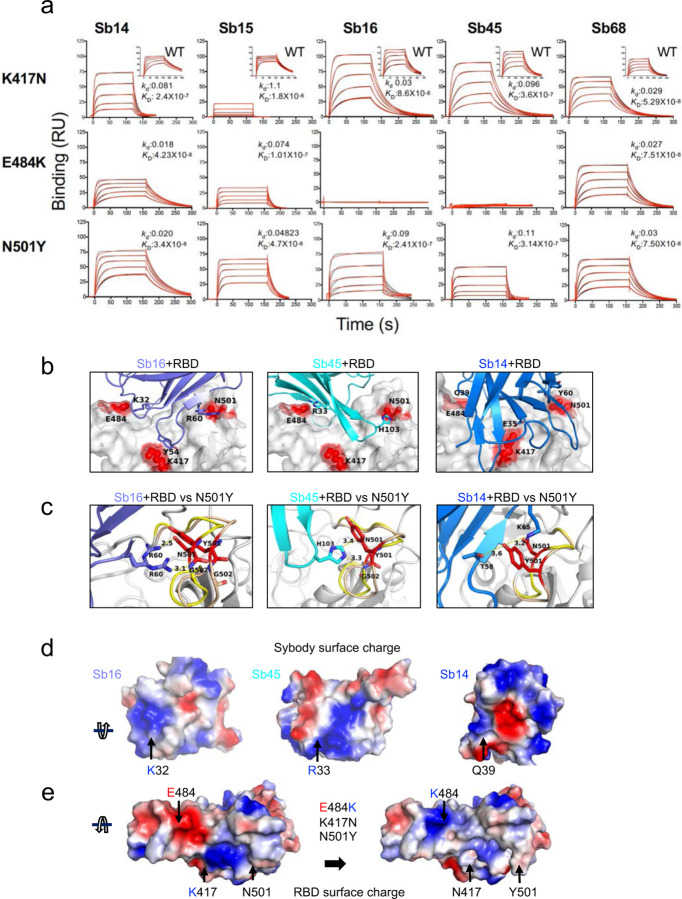
RBD mutations affect sybody binding. (**a**) SPR binding of each of the indicated sybodies (across top) to each of the individual RBD mutants. Inset shows binding of sybodies to wild type RBD (from Fig. 1). Experimental tracings are shown in red, curve fits in black and *k*_d_ (s^−1^) and *K*_D_ (M) values as determined from global fitting with BIAeval 2.0 are provided in each panel. (**b**) Location of contacts of Sb16, Sb45, and Sb14 are shown. E484, K417 and N501 of RBD (wild type) interact with K32, Y54 and R60 of Sb16 respectively; E484 and N501 of RBD (wild type) interact with R33 and H103 of Sb45 respectively; and E484, K417 and N501 of RBD (wild type) interact with Q39, E35, and Y60 of Sb14 respectively. (**c**) Comparison of complex structures with minimized models involving the N501Y mutation. *In* silico mutagenesis of N501Y was performed using 7KGK (Sb16+RBD), 7KGJ (Sb45+RBD), and 7MFU (Sb14+RBD+Sb68). Following amino acid substitution in Coot, local energy minimization (within 15 to 20 Å of the mutant residue) was performed through three rounds in PHENIX. For the Sb16-RBD complex, when N501 is mutated to Y501, the loop (496–506, from yellow to wheat) extends about 2.4 Å, but R60 (revealing a double conformation) still forms hydrogen bonds with the Y501 loop; for the Sb45-RBD complex, when N501 is mutated to Y501, the loop (496–506, from yellow to wheat) extends about 1.0 Å, but H103 of Sb45 would still interact with Y501; for the Sb14-RBD complex, when N501 is mutated to Y501, the loop (496–506, from yellow to wheat) is extended about 2.0 Å, but T58 and K65 still the hydrogen bonds with Y501; (**d**) The surface charge of Sb16, K32 forms a hydrogen bond with E484 of RBD with the opposite charge; the surface charge of Sb45, R33 forms a hydrogen bond with E484 of RBD with the opposite charge; the surface charge of Sb14, Q39 (a neutral residue) interacts with E484 of RBD; (**e**) Surface charge of wild type of RBD and surface charge of RBD with the three mutations (E484, K417N, and N501Y). When E484 is mutated to K484, the surface charge is changed from negative to positive, therefore the hydrogen bonds are broken – pushing Sb16 and Sb45 out of contact, while since Q39 of Sb14 is not a charged residue, it still may interact with K484 of the mutated RBD.

The major circulating variants, specifically B.1.1.7 (UK), B.1.351 (South Africa), and P.1 (Brazil), contain mutations in the RBD that lead to increased binding affinity to ACE2 and have the potential to reduce vaccine efficacy ^40–43^. Specifically, in addition to other mutations throughout the S protein and viral genome, all three harbor N501Y. B.1.351 and P.1 also have the E484K substitution, as well as substitution of K417 (to N for B.1.351 and to T for P.1). To assess the effect that substitution at each of these positions exerts on reactivity with Sb14, Sb15, Sb16, Sb45, and Sb68, we engineered individual mutations in the RBD and tested them by SPR (see Fig. 6a). In general, the five sybodies which interact with the parental (designated wild type (WT)) RBD with *K*_D_ values of 6.8 x 10^−9^ (for Sb15) to 6.3 x 10^−8^ M (for Sb68) (see Fig. 1), showed different patterns of binding to the K417N, E484K, and N501Y mutants. Sb68 bound each with similar affinity, consistent with its epitope lying outside of the ACE2 binding site on RBD, while each of the others revealed a distinct pattern. Sb14 binding was most affected by K417N. Sb15 bound both K417N and E484K less efficiently than N501Y. Sb16, largely unaffected in binding to K417N showed decreased recognition of N501Y and failed to interact detectably with E484K. Similar to Sb16, Sb45 also failed to bind E484K and showed decreased recognition of K417N and N501Y as compared to WT. To understand the structural basis of these differences in recognition of the different RBD mutants, we generated models based on the sybody–RBD structures (Fig. 6b–e). For Sb16, Sb45, and Sb14, interaction with the N501Y mutant resulted in displacement of its 496–506 loop by 2.0 Å, 1.0 Å, and 1.5 Å respectively. Nevertheless, R60 of Sb16 and H103 of Sb45 maintained contact with N501Y. This suggests that N501Y mutation would not escape recognition by these sybodies. Other cryo-EM studies_indicate modest effects of the N501Y substitution on binding to different antibodies ^44^. In contrast to the effects of N501Y, E484K revealed major incompatibilities due to charge repulsion, in the interaction with Sb16 via K32 and of Sb45 via R33 (Fig. 6d,e).

## Discussion

Our studies of the X-ray structures of Sb16 alone, Sb16–RBD, Sb45–RBD, the ternary Sb14– RBD–Sb68 and Sb45–RBD–Sb68 complexes, and the cryo-EM structures of Sb45–S provide critical detail describing the basis of the inhibition of S binding to the cell surface ACE2 receptor and the resulting block of viral infectivity. Sybodies and nanobodies, by virtue of their single domain structure and ability to be expressed in *E. coli* systems, as noted by others ^17,19^, offer advantages over Fab. Barnes et al ^45^ categorized a host of anti-S and anti-RBD Fabs into four classes (1–4) based on the location of the footprint, and whether the Fab has access to either the up only or up and down configuration of the RBD in the context of the full trimer (Extended Data Fig. 7a). By superposition (Extended Data Fig. 7a), Sb14 clearly belongs to Class 1 because it completely covers the light chain of the B38 Fab (7BZ5). Sb16 partially clashes with B38 but it primarily overlaps with the heavy chain of COVA2–39 (7JMP) and it can bind both to up and down positions of the RBD in S (Extended Data Fig.6), indicating that it belongs to Class 2 (Extended Data Fig. 7a). Sb45 clashes effectively with the heavy chain of COVA2–39, and our cryo-EM structures (7N0G, 7N0H) indicate that Sb45 can bind to both up and down forms of S-6P (Fig.5). Thus, Sb45 qualifies as Class 2 (Extended Data Fig. 7a). By contrast, Sb68 competes most with the CR3022 heavy chain (6W41), V_HH_72 (6WAQ) ^26^ and V_HH_-U (7KN5) ^46^ placing it in Class 4. However, unlike the other class 4 antibodies, Sb68 competes presumably due to its spatial orientation. Overall, our structural studies not only define the Sb14, Sb16, Sb45, and Sb68 epitopes at high resolution, but also reveal that these sybodies capture a rather large epitopic area (Supplementary Table 2), suggesting that a judicious choice of several sybodies or nanobodies have the potential to effectively saturate the available RBD surface.

The significance of the ternary structures of Sb45–RBD–Sb68 (7KLW) and Sb14–RBD– Sb68 (7MFU) is shown in a recent paper ^46^. Koenig et al ^46^ determined a ternary nanobody structure of V_HH_-E–RBD–V_HH_-U (7KN5) which illustrates the binding to two distinct epitopic sites. The ternary structure may also be considered as illustrative of the potential behavior of a bispecific construct linking two nanobodies. The bivalent or multivalent binding by antibody or nanobody would be expected to increase neutralization potential^19,46–48^. Superposition of Sb14–RBD–Sb68 or Sb45–RBD–Sb68 on V_HH_-E–RBD–V_HH_-U indicates that Sb14, Sb45 and V_HH_-E represent class 1 and class 2 in recognizing the epitopic region but do so in somewhat different orientations (Extended Fig. 7b). Sb45 exploits its two lengthy CDR2 and CDR3 loops which ride along both sides of the RBD surface, and Sb14 uses both CDR2 and CDR3 on the same side close to Sb68, while V_HH_-E uses a long CDR3 loop engaging one side of the RBD surface. Furthermore, Sb14 and Sb68 in Sb14–RBD–Sb68 (7MFU) show contacts (Y57-E44, G55-E44, and T54-H108) between two specific sybodies on the RBD surface (Extended Data Fig. 7c), which emphases the importance of bivalent and mutivalent antibodies/nanobodies against the virus.

Recently, several SARS-CoV-2 spike variants have been isolated and characterized with respect to their infectivity and severity of disease. The UK-SARS-CoV-2 variant has multiple substitutions including N501Y in the RBD ^1^. The mutation of E484K leads to repulsion of charged residues of antibody/nanobody/sybodies (Fig.6). To accommodate such a mutation, the complementary charged residues of the antibody/nanobody/sybody should also reverse their charge. Alternatively, employing another antibody/nanobody/sybody with opposite charge could capture such a escape mutation. Indeed, knowledge of the location of common or recurrent escape mutations and their potential resistance to antibody/nanobody/sybodies would provide a rational basis for either sequential or simultaneous use of reagents with complementary specificity. Thus, precise mapping of anti-RBD antibody, nanobody, and sybody epitopes, especially for those that are developed for clinical trials, has implications not only for mechanistic understanding of the interactions of the RBD with ACE2, but also for evaluating the potential susceptibility of newly arising viral variants to currently administered vaccines and antibodies.

## Online Methods

### Subcloning, expression and purification of RBD, spike, and sybody proteins.

The sequences encoding the RBD of the SARS-CoV-2 spike protein (amino acids 333 to 529) were subcloned into pET21b(+), (Novagen) via *Nde*I and *Eco*RI restriction sites, using pcDNA3-SARS-CoV-2-RBD-8his (Addgene #145145, ^49^) as template. The primers used were forward primer, 5’-TGCAGTCATATGAATCTTTGTCCGTTCGGTGAG and reverse primer, 5’-TGCAGTGAATTCTCACCCTTTTTGGGCCCACAAACT. The RBD was expressed as inclusion bodies in *E. coli* strain BL21(DE3) (Novagen). Expression, isolation of inclusion bodies, denaturation and reduction was done in 6 M guanidine hydrochloride and 0.1 mM DTT as described elsewhere ^50^. Briefly, refolding was carried out in a refolding buffer supplemented with oxidized and reduced glutathione and arginine for 3 days at 4 °C followed by dialysis against HEPES buffer (25 mM HEPES, pH 7.3, 150 mM NaCl). Concentrated and filtered protein was analyzed by size-exclusion chromatography on a Superdex 200 10/300 GL column (GE Healthcare) equilibrated with HEPES buffer. The peak corresponding to 24 kDa (monomeric) protein was collected, concentrated, and further purified by ion-exchange chromatography on Mono-Q® (Cytiva). Mutant RBD were generated by Site directed mutagenesis, performed with the QuikChange Lightning Multisite mutagenesis kit (Agilent, Santa Clara, CA, USA). All mutants were sequenced through GeneWiz and protein expression, refolding, and purification were done as described above.

Plasmids pSb-init encoding sybodies Sb14, Sb15, Sb16, Sb45. and Sb68 (Addgene #15322, 153523, #153524, #153526, and #153527, respectively) were originally reported by Walter et al ^18^ and generously made available. All plasmids were verified by DNA sequencing. Purification of the recombinant proteins from the periplasm of *E. coli* MC1061 was based on a protocol described elsewhere ^21^. Briefly, *E. coli* MC1061, transformed with a sybody-encoding plasmid, was grown in Terrific Broth (TB) medium (Gibco) supplemented with 25 μg/ml chloramphenicol, at 37 °C with shaking at 160 rpm for 2 hrs. The temperature was then decreased to 22 °C until A600 reached 0.5. Protein expression was induced by addition of L-(+)-arabinose (Sigma) to a final concentration of 0.02% (w/v) and expression continued overnight at 22 °C and 160 rpm. The next day cells were collected by centrifugation at 2000 x *g* for 15 minutes. The cell pellet was then washed twice in PBS and resuspended in periplasmic extraction buffer (50 mM Tris/HCl pH 8.0, 0.5 mM EDTA, 0.5 μg/ml lysozyme, 20% w/v sucrose (Sigma)) at 4 °C for 30 min followed by addition of TBS (pH 8.0) and 1 mM MgCl2. Cells were then centrifuged at 10,000 rpm (Fiberlite™ F21–8 x 507 Fixed Angle Rotor) for 30 min. Following transfer of the supernatant to a fresh tube, imidazole was added to a final concentration of 10 mM. Ni-NTA resin (Qiagen) equilibrated with TBS was added to the supernatant and incubated for 1 hr at RT with mild agitation. The resin was collected, washed three times with buffer supplemented with 30 mM imidazole and sybody proteins were eluted with 300 mM imidazole in TBS.

Plasmid encoding spike HexaPro (designated “S” throughout) was procured from Addgene (#154754) ^33^ and transfected into Expi293F™ cells (ThermoFisher Scientific) using manufacturer’s protocol. Briefly, Expi293F™ cells were seeded to a final density of 2.5–3 × 10^6^ viable cells/ml and grown overnight at 37 °C in Expi293™ Expression Medium (Gibco). The following day, cell viability was determined, and cell density was adjusted to 3 × 10^6^ viable cells/ml with fresh, prewarmed Expi293™ Expression Medium. Transfection was then done as per manufacturer’s instructions using 1 μg/ml plasmid DNA. Cultures were grown for 6 days following transfection and supernatant was collected, filtered through a 0.22 µm filter and passed over Ni-NTA resin for affinity purification. Further purification was accomplished by size-exclusion chromatography using a Superose 6 10/300 GL column (Cytiva) in a buffer consisting of 2 mM Tris pH 8.0, 200 mM NaCl. Purification of sybodies, RBD, and 6-SP is shown in Extended Data Fig.8.

### Preparative and analytical size-exclusion chromatography.

Sybodies purified by Ni-NTA affinity chromatography were concentrated using Amicon 10K MWCO concentrators and purified on a Sepax SRT-10C SEC100 column at a flow rate of 1 ml/min. Monomeric sybodies elute at a retention volume of 11–12.5 ml from the Sepax SRT-10C SEC100 column. Monomeric peak fractions were collected and analyzed by SDS-PAGE. Analytical SEC of RBD-sybody complexes was performed on a Shodex KW-802.5 column at a flow rate of 0.75 ml/min in TBS buffer (pH. 8.0). (The interaction of individual sybodies with the column matrix is a well-documented phenomenon ^21^).

### Surface Plasmon Resonance.

SPR experiments were performed on a Biacore T200 (Cytiva) at 25 °C in 10 mM HEPES pH 7.2, 150 mM NaCl, 3 mM EDTA, 0.05% Tween-20. RBD was immobilized on a series S CM5 sensor chip (Cytiva) by amine (NHS/EDC) coupling to flow cells. For background subtraction a reference cell was mock coupled. Binding and kinetic studies were performed multiple times for each sybody. Graded and increasing concentrations of SB16, SB45 and SB68 were injected over the RBD-immobilized surface at a flow rate of 30ml/min with an association time of 120 s and dissociation time of 2000 s. Binding data were analyzed by surface site affinity distribution analysis by EVILFIT ^51,52^. In general, these values were consistent with fits to the Langmuir binding equation for a 1:1 interaction model using Biacore T200 Evaluation Software v3.0, but revealed better statistics.

### Thermal stability.

Thermal melt analysis of the recombinant proteins was performed in triplicate in 96-well plates in a QuantStudio 7 Flex real time PCR machine (Applied Biosystems). Each well contained 2–4 mg protein in buffer (25mM TRIS pH 8, 150 mM NaCl) and 5x Sypro Orange (Invitrogen, stock 5000x) in a total volume of 20 ml. Following an initial two-minute hold at 25 °C, the plate was heated to 99 °C at a rate of 0.05 °C/sec. Data were analyzed with Protein Thermal Shift Software v1.3 (Invitrogen) to obtain T_m_ values for RBD, S, Sb14, Sb15, Sb16, Sb45, and Sb68 (Extended Data Fig. 9).

### Crystallization, data collection, structure determination and crystallographic refinement.

Purified sybodies (Sb14, Sb15, Sb16, Sb45 and Sb68) and RBD were mixed in approximate 1:1 molar ratio to a final concentration of 8 mg/ml. The protein mixtures were incubated on ice for 1 hour prior to screening. Initial screening for crystals was carried out using the hanging drop vapor diffusion method using the Mosquito robotic system (sptlabtech.com). Crystals of SB16–RBD and SB45-RBD complexes and Sb16 alone were observed within one week using Protein Complex (Qiagen) and Wizard Classic 4 (Rigaku). Conditions for Sb16–RBD were either 0.1M HEPES pH 7.0, 15% PEG 20000, or 0.1M HEPES pH 7.0, 18% PEG 12000; and for Sb45–RBD was 18% PEG 12000 and 12% PEG 8000, 0.1 M HEPES pH 7.5, 0.2 M NaCl. Crystallization condition for Sb14–RBD–Sb68 was 12% PEG 8000, 0.1 M MOPS, pH 7.5, 0.1 M Mg Acetate. Sb16 alone crystallized in 20% PEG 4000, 0.1 M MES, pH 6.0, 0.2 M LiSO_4_. We also screened mixtures of two or three sybodies with RBD. Crystals of Sb45–RBD–Sb68 were obtained after one month following mixing the three proteins in an equimolar ratio in 10% PEG 8000, 0.1M sodium cacodylate pH 6.0.

Crystals of protein complexes were optimized with slight adjustments of the concentration of PEG components. Crystals were cryoprotected in mother liquor containing 5% ethylene glycol and 5% glycerol and flash frozen in liquid nitrogen for data collection. Diffraction data were collected at the Southeast Regional Collaborative Access Team (SER-CAT) beamline 22ID-D at the Advanced Photon Source, Argonne National Laboratory and data were processed with XDS ^53^. Multiple data sets were collected for the protein complexes from 2.3–3.2 Å resolution. The initial model of Sb16 and Sb45 for the molecular replacement search were built by the MMM server (manaslu.fiserlab.org/MMM ^54^), using the heavy chain V domain and the RBD of the Fab B38–RBD complex (PDBid: 7BZ5) ^22^. The initial model of Sb68 for molecular replacement was built based on the V_H_ domain of 7BZ5. Molecular replacement solutions were found using Phaser ^39,55^. Subsequent refinements were carried out with PHENIX ^56^. CDR loops were manually rebuilt by fitting to the electron density maps with Coot ^57^. In particular, Sb68 CDR loops were deleted before refinement and built in manually based on electron density maps. Illustrations and calculations of superpositioned models were prepared in PyMOL (The PyMOL Molecular Graphics System, Version 2.4.0 Schrödinger, LLC). Calculation of hinge relationships of domains was accomplished with HINGE (https://collab.niaid.nih.gov/sites/research/DIR/LIG/SIS/Lists/Programs/homeview.aspx) provided courtesy of Peter Sun, NIAID. Buried surface area (BSA) calculations were performed with PISA (https://www.ebi.ac.uk/pdbe/pisa/). The final structures for the RBD-SB16 and RBD-SB45 complexes showed *R*_work_/*R*_free_ (%) 25.4/27.7 and 18.6/21.6 respectively, and for SB16 alone with *R*_work_/*R*_free_ 22.4/25.9. Data collection and structure refinement statistics are provided in Table 1.

### Cryo-EM sample preparation and data collection.

Freshly purified S-6P was incubated with Sb45 in a 1:3 molar ratio and repurified by size exclusion chromatography. Negative stain screening was accomplished with a Tecnei T12 120-keV microscope (Thermo Fisher). The protein complexes were concentrated to 0.7–1 mg/ml and 3 μl of the sample was applied onto holey-carbon cryo-EM grids (Cu R1.2/1.3, 300 mesh, Quantifoil), which had been glow discharged for 60 seconds, blotted for 3 seconds, and plunge frozen into liquid ethane with a Vitrobot (Thermo Fisher Scientific) at 4 °C and 95% humidity. Cryo-EM data in selected grid regions were collected on a Titan Krios 300-keV microscope (Thermo Fisher). Images were acquired automatically with SerialEM ^58^ on a BioQuantum-K2 summit detector (Gatan) with a 20eV energy filter slit in super-resolution mode at 130x nominal magnification (1.052 Å binned pixel size) and a defocus range from −0.7 to −2.0 μm. An exposure time of 8s at 0.2s per frame was recorded with a total exposure of about 56 electrons/Å^2^. Two raw data sets were collected on two frozen grids: one with 1,780 micrographs and one with 7,945 micrographs.

### Image processing and structure solution.

All image processing, 2D class, 3D reconstruction, and map refinements were performed with cryoSPARC v3.1 and v3.2 ^36 34,35,37^. A total of 9,725 micrographs was imported into cryoSPARC. Following “patch motion correction” and “patch CTF estimation,” the number of micrographs was reduced to 9,703. Micrographs were inspected by “curate exposures,” in which outliers of defocus range, defective micrographs, and those with a low-resolution estimation of the CTF fit (>5 Å) were discarded, resulting in 9,237 micrographs. “Blob picker” was used with the particle diameter between 128 and 256 angstroms for picking particles. After “inspect particles” with NCC (Normalized Correlation Coefficient) 0.28 and “power threshold” between 500 and 1000 (which removed ice and aggregates), the number of particles was 1,876,941. To determine the “box size,” we performed several trials indicating that the box size should be larger than 336 pixels, and finally used a box size of 400 pixels and extracted 1,433,963 particles. After “2D classification” (100 classes), 18 2D classes were selected, retaining 662,994 particles. The particles were submitted to a series of “Ab initio 3D reconstruction” classification and divided into 2 or 4 sub-groups. After removing the particles of un-recognized or “defective” shape, a total of 417,460 particles with shape resembling spike remained. These particles were subjected to “homogeneity refinement,” followed by “CTF global and local refinement” and “non-uniform refinement.” No symmetry was imposed aside from C1 during the map refinements. The map after refinement could reach 2.84 Å resolution by the gold-standard FSC estimation with a 0.143 cut-off criterion. We then identified further the two conformations of S-6P as previously described ^33^. One sub-class of 214,171 particles revealed the conformation of “1-up, 2-down” of RBD (Extended Data Fig. 4c), and one sub-class of 61,062 particles showed the conformation of “2-up, 1-down” (Extended Data Fig. 4c). The maps of “1-up,2-down” and “2-up,1-down” were refined at 3.02 Å and 3.34 Å resolution respectively. Local resolution plots for each map are shown in Extended Data Fig. 4d,e. The maps are deposited in EMDB as EMD-24105 and EMD-24106.

An initial model for S-6P was generated using PDB 6XKL and was fit as a rigid body into the map using Chimera ^38^ followed by PyMOL. The Sb45–RBD (7KGJ) crystal structure was superimposed onto the S-6P model in PyMOL. We used real space refinement in PHENIX ^39^ including rigid-body refinement. The model was split into subdomains, NTD (24–289) and RBD (334–528) for rigid-body refinement. Simulated annealing (SA) was performed initially, including a local grid search and ADP refinement, using secondary structure restraints. We noticed that the original 6XKL model lacked some loops in RBD and NTD domains, which were replaced by the RBD domain from 7KGJ, and the NTD domain from 7B62 ^59^ with all loops. For the model of the “1-up” form of S-6P, the CC was 0.84/0.78 (volume/peaks) with three Sb45 domains bound to three RBDs. However, the CC for three Sb45-X, Sb45-Y, and Sb45-Z are 0.51, 0.49, and 0.58 respectively, which indicates that the Sb45 does not fully bind to S-6P. For the “2-up” form of S-6P, we first generated the model by superimposing the A-chain of the “1-up” form of S-6P onto B-chain and replaced B-chain for the real space refinement, the resulting model with an overall CC of 0.83/0.76 (volume/peaks), but with only two Sb45 domains, one Sb45-X binds to A-chain (up RBD) and one Sb45-Z binds to C-chain (down RBD) with CC 0.44 and 0.68 respectively. These two models are deposited in PDB as 7N0G and 7N0H. Data processing, refinement statistics, and model validation are listed in Table 2.

## Supplementary Material

Supplement 1

## Figures and Tables

**Table 1|:** X-ray data collection and refinement statistics

	Sb16-RBD	Sb45-RBD	Sb14-RBD-Sb68	Sb45-RBD-Sb68	Sb16

PDBID	7KGK	7KGJ	7MFU	7KLW	7MFV
**Data collection**					
Space group	P6_5_22	P3_2_21	P2_1_	C222_1_	P6_3_22
Cell dimensions					
*a*, *b*, *c* (Å)*	65.64, 65.64, 344.69	62.55, 62.55, 168.82	66.82, 83.05, 92.83	74.50, 102.40, 138.97	68.92, 68.92, 107.17
α*, β,* γ (°)	90.0, 90.0, 120.0	90.0, 90.0, 120.0	90.0, 106.71, 90.0	90.0, 90.0, 90.0	90.0, 90.0, 120.0
Resolution (Å)	57.34–2.60 (2.69–2.60)	45.59–2.30 (2.38–2.30)	42.17–1.70 (1.76–1.70)	44.12–2.60 (2.69–2.60)	34.46–1.90 (1.97–1.90)
*R*_*sym*_ *or R*_*merge*_	0.080 (0.455)	0.101 (0.849)	0.086 (0.765)	0.095 (0.739)	0.074 (3.39)
*I/*σ*(I)*	18.0 (3.3)	14.9 (3.4)	8.9 (1.7)	13.1 (2.1)	15.2 (0.7)
Completeness (%)	98.8 (99.1)	99.3 (98.3)	98.4 (93.8)	98.8 (98.7)	94.5 (85.0)
Redundancy	10.3 (10.9)	7.9 (8.2)	3.1 (3.1)	7.2 (7.4)	12.4 (12.6)
*R*_*pim*_	0.024 (0.134)	0.038 (0.293)	0.057 (0.510)	0.038 (0.287)	0.022 (1.05)
CC_1/2_	0.999 (0.987)	0.997 (0.919)	0.995 (0.640)	0.998 (0.895)	0.999 (0.526)
Estimated twin fraction	0.0 (none)	0.06 (−h, −k, l)	0.0 (none)	0.0 (none)	0.0 (none)
**Refinement**					
Resolution (Å)	56.09–2.60 (2.69–2.60)	45.59–2.30 (2.38–2.30)	42.27–1.70 (1.76–1.70)	36.72–2.60 (2.69–2.60)	34.46–1.90 (1.97–1.90)
No. reflections	13219 (1185)	17592 (1687)	105129 (9993)	16508 (1627)	11788 (1025)
*R*_work_/*R*_free_ (%)	25.8/27.7 (36.3/44.2)	18.6/21.6 (24.1/29.8)	18.1/21.5 (27.0/31.6)	20.6/25.5 (29.3/34.5)	22.8/25.8 (34.5/33.5)
No. atoms	2486	2641	7798	3552	976
Protein	2486	2500	6798	3456	907
Water + ligands	0	141	962+38	96	65+4
B-factor Wilson/Ave	39.3/59.8	26.9/32.9	20.3/26.9	33.9/31.5	31.8/45.0
Protein	59.8	32.8	25.7	31.5	45.0
Water + ligands	0	34.7	34.5+40.0	29.5	45.1+55.8
R.m.s. deviations					
bond length (Å)	0.002	0.005	0.004	0.003	0.013
bond angle (°)	0.54	0.74	0.74	0.64	1.17
Ramachandran					
favored (%)	92.9	97.4	98.3	96.3	95.6
allowed (%)	7.1	2.6	1.5	3.7	3.5
outliers (%)	0.0	0.0	0.2	0.0	0.9

* Values in parenthesis are for highest resolution shell.

**Table 2 |:** Cryo-EM data collection, refinement and validation statistics

	Sb45+S-6P (1-up,2-down)	Sb45+S-6P (2-up,1-down)
EMDB ID	EMD-24105	EMD-24106

PDB-ID	7N0G	7N0H

Data collection and processing		
Magnification	130,000	130,000
Voltage (kV)	300	300
Electron exposure (e^−^/Å)	56	56
Defocus range (μm)	−0.7 to −2.0	−0.7 to −2.0
Pixel size (Å/pixel)	0.526 (1.052 binned)	0.526 (1.052 binned)
Raw micrographs (no.)	9,725	9,725
Extract particles (no.)	1,447,993	1,447,993
Selected 2D particles (no.)	662,994	662,994
Refined particles (no.)	417,460	417,460
Particles for final map (no.)	214,171	60,062
Symmetry imposed	C1	C1
Map resolution (Å)	3.02	3.34
FSC threshold	0.143	0.143
Refinement		
Initial model used	6XKL, 7KGJ	6XKL, 7KGJ
Model composition		
Atoms	29,062	27,974
Residues	3,592	3,469
Ligands (NAG)	73	64
Overall B-factor (Å^2^)		
Protein (min/max/mean)	36.8/589.6/157.0	24.2/485.3/157.0
Ligands (min/max/mean)	55.3/340.1/129.9	51.8/358.8/144.5
R.m.s. deviations		
bond length (Å)	0.003	0.005
bond angle (°)	0.548	0.972
CC (mask/volume/peaks)	0.84/0.84/0.77	0.83/0.83/0.77
Validation		
MolProbity score	1.62	1.71
Clashscore	7.71	8.26
Poor rotamers	0.00	0.00

## References

[1] Conti P. The British variant of the new coronavirus-19 (Sars-Cov-2) should not create a vaccine problem. J Biol Regul Homeost Agents 35(2021). 33377359

[2] Kirby T. New variant of SARS-CoV-2 in UK causes surge of COVID-19. Lancet Respir Med (2021). 10.1016/S2213-2600(21)00005-9 PMC7784534 33417829

[3] Tang J.W., Tambyah P.A. & Hui D.S. Emergence of a new SARS-CoV-2 variant in the UK. J Infect (2020). 10.1016/j.jinf.2020.12.024 PMC7834693 33383088

[4] Wibmer C.K. SARS-CoV-2 501Y.V2 escapes neutralization by South African COVID-19 donor plasma. bioRxiv, 2021.01.18.427166 (2021). 10.1038/s41591-021-01285-x 33654292

[5] Wang Q. Structural and Functional Basis of SARS-CoV-2 Entry by Using Human ACE2. Cell (2020). 10.1016/j.cell.2020.03.045 PMC7144619 32275855

[6] Shang J. Cell entry mechanisms of SARS-CoV-2. Proc Natl Acad Sci U S A 117, 11727–11734 (2020). 32376634 10.1073/pnas.2003138117 PMC7260975

[7] Baden L.R. Efficacy and Safety of the mRNA-1273 SARS-CoV-2 Vaccine. N Engl J Med (2020). 10.1056/NEJMoa2035389 PMC7787219 33378609

[8] Cao Y. Potent Neutralizing Antibodies against SARS-CoV-2 Identified by High-Throughput Single-Cell Sequencing of Convalescent Patients’ B Cells. Cell 182, 73–84 e16 (2020). 32425270 10.1016/j.cell.2020.05.025 PMC7231725

[9] Cerutti G. Structural Basis for Accommodation of Emerging B.1.351 and B.1.1.7 Variants by Two Potent SARS-CoV-2 Neutralizing Antibodies. bioRxiv (2021). 10.1016/j.str.2021.05.014 PMC8188728 34111408

[10] Edara V.V. Reduced binding and neutralization of infection- and vaccine-induced antibodies to the B.1.351 (South African) SARS-CoV-2 variant. bioRxiv (2021).

[11] Planas D. Sensitivity of infectious SARS-CoV-2 B.1.1.7 and B.1.351 variants to neutralizing antibodies. Nat Med (2021). 10.1038/s41591-021-01318-5 33772244

[12] Ramanathan M., Ferguson I.D., Miao W. & Khavari P.A. SARS-CoV-2 B.1.1.7 and B.1.351 Spike variants bind human ACE2 with increased affinity. bioRxiv (2021). 10.1016/S1473-3099(21)00262-0 PMC8133765 34022142

[13] Singh J. Structure-Function Analyses of New SARS-CoV-2 Variants B.1.1.7, B.1.351 and B.1.1.28.1: Clinical, Diagnostic, Therapeutic and Public Health Implications. Viruses 13(2021). 10.3390/v13030439 PMC8000172 33803400

[14] Wang P. Antibody resistance of SARS-CoV-2 variants B.1.351 and B.1.1.7. Nature (2021). 10.1038/s41586-021-03398-2 33684923

[15] Xu C. Conformational dynamics of SARS-CoV-2 trimeric spike glycoprotein in complex with receptor ACE2 revealed by cryo-EM. Sci Adv (2020). 10.1126/sciadv.abe5575 PMC7775788 33277323

[16] Hanke L. An alpaca nanobody neutralizes SARS-CoV-2 by blocking receptor interaction. Nat Commun 11, 4420 (2020). 32887876 10.1038/s41467-020-18174-5 PMC7473855

[17] Schoof M. An ultrapotent synthetic nanobody neutralizes SARS-CoV-2 by stabilizing inactive Spike. Science (2020). 10.1126/science.abe3255 PMC7857409 33154106

[18] Walter J.D. Highly potent bispecific sybodies neutralize SARS-CoV-2. bioRxiv (2020).

[19] Xiang Y. Versatile and multivalent nanobodies efficiently neutralize SARS-CoV-2. Science 370, 1479–1484 (2020). 33154108 10.1126/science.abe4747 PMC7857400

[20] Ingram J.R., Schmidt F.I. & Ploegh H.L. Exploiting Nanobodies’ Singular Traits. Annu Rev Immunol 36, 695–715 (2018). 29490163 10.1146/annurev-immunol-042617-053327

[21] Zimmermann I. Generation of synthetic nanobodies against delicate proteins. Nat Protoc 15, 1707–1741 (2020). 32269381 10.1038/s41596-020-0304-x PMC7617899

[22] Wu Y. A noncompeting pair of human neutralizing antibodies block COVID-19 virus binding to its receptor ACE2. Science 368, 1274–1278 (2020). 32404477 10.1126/science.abc2241 PMC7223722

[23] Natarajan K., Mage M.G. & Margulies D.H. Immunoglobulin Superfamily. in eLS (John Wiley & Sons, Ltd, Chichester, 2015).

[24] Halaby D.M., Poupon A. & Mornon J. The immunoglobulin fold family: sequence analysis and 3D structure comparisons. Protein Eng 12, 563–71 (1999). 10436082 10.1093/protein/12.7.563

[25] Huo J. Neutralization of SARS-CoV-2 by Destruction of the Prefusion Spike. Cell Host Microbe 28, 445–454 e6 (2020). 32585135 10.1016/j.chom.2020.06.010 PMC7303615

[26] Wrapp D. Structural Basis for Potent Neutralization of Betacoronaviruses by Single-Domain Camelid Antibodies. Cell 181, 1004–1015 e15 (2020). 32375025 10.1016/j.cell.2020.04.031 PMC7199733

[27] Wu T.T. & Kabat E.A. An analysis of the sequences of the variable regions of Bence Jones proteins and myeloma light chains and their implications for antibody complementarity. J Exp Med 132, 211–50 (1970). 5508247 10.1084/jem.132.2.211 PMC2138737

[28] Lan J. Structure of the SARS-CoV-2 spike receptor-binding domain bound to the ACE2 receptor. Nature 581, 215–220 (2020). 32225176 10.1038/s41586-020-2180-5

[29] Rudd P.M. Hybrid and complex glycans are linked to the conserved N-glycosylation site of the third eight-cysteine domain of LTBP-1 in insect cells. Biochemistry 39, 1596–603 (2000). 10677208 10.1021/bi9918285

[30] Hsu T.A. Differential N-glycan patterns of secreted and intracellular IgG produced in Trichoplusia ni cells. J Biol Chem 272, 9062–70 (1997). 9083032 10.1074/jbc.272.14.9062

[31] Shajahan A. Comprehensive characterization of N- and O- glycosylation of SARS-CoV-2 human receptor angiotensin converting enzyme 2. Glycobiology (2020). 10.1093/glycob/cwaa101 PMC7665489 33135055

[32] Zhao P. Virus-Receptor Interactions of Glycosylated SARS-CoV-2 Spike and Human ACE2 Receptor. Cell Host Microbe 28, 586–601 e6 (2020). 32841605 10.1016/j.chom.2020.08.004 PMC7443692

[33] Hsieh C.L. Structure-based design of prefusion-stabilized SARS-CoV-2 spikes. Science 369, 1501–1505 (2020). 32703906 10.1126/science.abd0826 PMC7402631

[34] Punjani A. & Fleet D.J. 3D Variability Analysis: Resolving continuous flexibility and discrete heterogeneity from single particle cryo-EM. BioRXiv, bioRxiv 2020.04.08.032466 (2020). 10.1016/j.jsb.2021.107702 33582281

[35] Punjani A. & Fleet D.J. 3D Flexible Refinement: Structure and Motion of Flexible Proteins from Cryo-EM. BioRxiv, bioRxiv 2021.04.22.440893 (2021). 10.1038/s41592-023-01853-8 PMC10250194 37169929

[36] Punjani A., Rubinstein J.L., Fleet D.J. & Brubaker M.A. cryoSPARC: algorithms for rapid unsupervised cryo-EM structure determination. Nat Methods 14, 290–296 (2017). 28165473 10.1038/nmeth.4169

[37] Punjani A., Zhang H. & Fleet D.J. Non-uniform refinement: Adaptive regularization improves single particle cryo-EM reconstruction. BioRXiv, bioRxiv 2019.12.15.877092 (2019). 10.1038/s41592-020-00990-8 33257830

[38] Pettersen E.F. UCSF Chimera--a visualization system for exploratory research and analysis. J Comput Chem 25, 1605–12 (2004). 15264254 10.1002/jcc.20084

[39] Adams P.D. PHENIX: a comprehensive Python-based system for macromolecular structure solution. Acta Crystallogr D Biol Crystallogr 66, 213–21 (2010). 20124702 10.1107/S0907444909052925 PMC2815670

[40] Boehm E. Novel SARS-CoV-2 variants: the pandemics within the pandemic. Clin Microbiol Infect (2021). 10.1016/j.cmi.2021.05.022 PMC8127517 34015535

[41] Wang Z. mRNA vaccine-elicited antibodies to SARS-CoV-2 and circulating variants. Nature 592, 616–622 (2021). 33567448 10.1038/s41586-021-03324-6 PMC8503938

[42] Wibmer C.K. SARS-CoV-2 501Y.V2 escapes neutralization by South African COVID-19 donor plasma. Nat Med 27, 622–625 (2021). 33654292 10.1038/s41591-021-01285-x

[43] Starr T.N. Prospective mapping of viral mutations that escape antibodies used to treat COVID-19. Science 371, 850–854 (2021). 33495308 10.1126/science.abf9302 PMC7963219

[44] Zhu X. Cryo-electron microscopy structures of the N501Y SARS-CoV-2 spike protein in complex with ACE2 and 2 potent neutralizing antibodies. PLoS Biol 19, e3001237 (2021). 33914735 10.1371/journal.pbio.3001237 PMC8112707

[45] Barnes C.O. SARS-CoV-2 neutralizing antibody structures inform therapeutic strategies. Nature (2020). 10.1038/s41586-020-2852-1 PMC8092461 33045718

[46] Koenig P.A. Structure-guided multivalent nanobodies block SARS-CoV-2 infection and suppress mutational escape. in Science 2021/01/14 edn (2021). 10.1126/science.abe6230 PMC7932109 33436526

[47] Xu J. Multimeric nanobodies from camelid engineered mice and llamas potently neutralize SARS-CoV-2 variants. bioRxiv (2021). 10.1038/s41586-021-03676-z PMC8260353 34098567

[48] Hansen J. Studies in humanized mice and convalescent humans yield a SARS-CoV-2 antibody cocktail. Science 369, 1010–1014 (2020). 32540901 10.1126/science.abd0827 PMC7299284

[49] Chan K.K. Engineering human ACE2 to optimize binding to the spike protein of SARS coronavirus 2. Science 369, 1261–1265 (2020). 32753553 10.1126/science.abc0870 PMC7574912

[50] Li H., Natarajan K., Malchiodi E., Margulies D. & Mariuzza R. Three-dimensional structure of H-2D(d) complexed with an immunodominant peptide from human immunodeficiency virus envelope glycoprotein 120. Journal of Molecular Biology 283, 179–191 (1998). 9761682 10.1006/jmbi.1998.2091

[51] Zhao H., Boyd L.F. & Schuck P. Measuring Protein Interactions by Optical Biosensors. Curr Protoc Protein Sci 88, 20 2 1–20 2 25 (2017). 28369667 10.1002/cpps.31 PMC5776739

[52] Zhao H., Gorshkova II, Fu G.L. & Schuck P. A comparison of binding surfaces for SPR biosensing using an antibody-antigen system and affinity distribution analysis. Methods 59, 328–35 (2013). 23270815 10.1016/j.ymeth.2012.12.007 PMC3840496

[53] Kabsch W. Xds. Acta Crystallogr D Biol Crystallogr 66, 125–32 (2010). 20124692 10.1107/S0907444909047337 PMC2815665

[54] Rai B.K. & Fiser A. Multiple mapping method: a novel approach to the sequence-to-structure alignment problem in comparative protein structure modeling. Proteins 63, 644–61 (2006). 16437570 10.1002/prot.20835

[55] McCoy A.J. Phaser crystallographic software. J Appl Crystallogr 40, 658–674 (2007). 19461840 10.1107/S0021889807021206 PMC2483472

[56] Liebschner D. Macromolecular structure determination using X-rays, neutrons and electrons: recent developments in Phenix. Acta Crystallogr D Struct Biol 75, 861–877 (2019). 31588918 10.1107/S2059798319011471 PMC6778852

[57] Emsley P., Lohkamp B., Scott W.G. & Cowtan K. Features and development of Coot. Acta Crystallogr D Biol Crystallogr 66, 486–501 (2010). 20383002 10.1107/S0907444910007493 PMC2852313

[58] Mastronarde D.N. Automated electron microscope tomography using robust prediction of specimen movements. J Struct Biol 152, 36–51 (2005). 16182563 10.1016/j.jsb.2005.07.007

[59] Custodio T.F. Selection, biophysical and structural analysis of synthetic nanobodies that effectively neutralize SARS-CoV-2. Nat Commun 11, 5588 (2020). 33149112 10.1038/s41467-020-19204-y PMC7642358

