# Supplementary material for: Structures of synthetic nanobody–SARS-CoV-2–RBD complexes reveal distinct sites of interaction and recognition of variants
Source: Res Sq. 2021 Jun 16:rs.3.rs-625642. Preprint. [Version 1] doi: 10.21203/rs.3.rs-625642/v1 (PMC8219104; doi:10.21203/rs.3.rs-625642/v1)
Supplement: Supplement 1 [file 53ac16e4a807768f05982382.pdf]

Supplementary Table 1 | Sybody contacts to RBD

RBD residue	Sb14 contact	Dist (Å)	Sb14 contact	Dist (Å)	Region
R403	E35	2.97	W47	3.30	beta3
D405	K52	2.62	T56	3.15	CDR2
G416	Y99	3.76			CDR3
K417	E35	2.89	Y37	3.69	beta3
D420	Y99	2.61			CDR3
Y421	S103	3.38	S102	3.58	CDR3
G446	Y62	3.51			loop5
Y453	Y37	2.68			beta3
L455	S103	2.62	Y97	3.49	CDR3
F456	R45	3.79			beta4
E484	Q39	2.89			beta3
N487	Q107	3.61			beta9
Y489	I105	3.52			beta9
F490	R45	2.81			beta4
L492	R45	3.26			beta4
Q493	R45	2.89	W47	3.52	beta4
S494	E44	3.47			beta4
Q498	Y60	2.82	Y62	3.48	beta5
T500	K65	3.07			loop5
N501	Y60	2.84	A59	3.52	beta5
G502	T56	3.74			CDR2
V503	T56	3.67			CDR2
G504	T56	2.91			CDR2
Y505	E33	2.62	K52	3.26	CDR1

RBD residue	Sb16 contact	Dist (Å)	Sb16 contact	Dist (Å)	Region
R403	Y54	3.88			CDR2
E406	Y54	2.65			CDR2
K417	Y54	3.42			CDR2
V445	R45	3.72			beta4
G446	Y37	3.23			CDR1
G447	Y37	3.39			CDR1
Y449	I98	3.51	W35	3.40	CDR3
L452	W100	3.45			CDR3
Y453	S53	2.64	Y54	3.71	CDR2
L455	Y31	3.48	Y54	3.39	CDR1
F456	Y31	3.32			CDR1
E484	K32	2.56	P28	3.47	CDR1
G485	P28	3.43			CDR1
Y489	A30	3.55	Y31	3.45	CDR1
F490	W100	3.75			CDR3
L492	W100	3.45			CDR3
Q493	W100	3.05	T33	3.17	CDR3
S494	T33	3.78	W100	3.62	CDR1
Y495	W35	3.23			CDR2
G496	W35	3.60			CDR1
Q498	W47	3.19	Y37	3.28	Beta3&4
N501	R60	3.49			CDR2
G502	R60	2.94			CDR2
Y505	E52	3.61	R60	3.55	CDR2

RBD residue	Sb45 contact	Dist (Å)	Sb45 contact	Dist (Å)	Region
Y351	A54	3.53			CDR2
R403	H103	2.76	G102	3.06	CDR3
D405	Y105	2.92			CDR3
G446	F27	3.58	G26	3.51	CDR1
G447	P28	3.28			CDR1
Y449	D100	3.07	R31	3.62	CDR3/1
N450	Y30	3.27			CDR1
L452	S53	3.80	D32	3.76	CDR2
Y453	Y107	3.03	V101	3.64	CDR3
L455	Y107	3.05			CDR3
T470	R59	2.84	G55	3.18	CDR2
I472	R59	3.67			CDR1
G482	R59	3.23			CDR1
V483	Y60	3.62	K65	3.37	CDR2
E484	R33	2.96	Y52	2.44	CDR1
F490	Y52	3.48			CDR2
L492	A54	3.74			CDR2
Q493	K99	2.96	D32	3.39	CDR3
S494	D32	2.63			CDR1
Q498	G26	3.14			CDR1
N501	H103	3.50			CDR3
G502	H103	3.32			CDR3
Y505	V101	3.25	H103	3.75	CDR3

RBD residue	Sb68 contact	Dist (Å)	Sb68 contact	Dist (Å))	Region
Y369	Y103	2.82	T32	3.43	CDR3/1
N370	N55	2.93	V54	3.56	CDR2
S371	H57	3.77			CDR2
F374	Y59	2.98	Y103	3.54	CDR2/3
S375	W105	2.80	A104	3.34	CDR3
T376	Y103	3.47	A104	3.74	CDR3
F377	Y103	2.86	G102	3.31	CDR3
K378	D111	2.84	W101	3.33	CDR3
C379	W101	2.87			CDR3
P384	G102	3.62	A100	3.72	CDR3
R408	D111	3.06	D110/H108	3.39/3.42	CDR3

Supplementary Table 2. Interface Buried Surface Area of Sybody/Nanbody/Fab with RBD/Spike

(Values of buried surface area (BSA) are shown. N indicates number of residues in contact, N_{HB} is number of hydrogen bonds, N_{SB} is number of salt bridge. Data were calculated from indicated PDB by PISA: https://www.ebi.ac.uk/pdbe/pisa/)

Complex(A+B)	BSA ($Å^2$)	$_AN_{res}$	$_BN_{res}$	N_{HB}	N_{SB}	PDB	Resol
ACE2+RBD	**844**	26	26	13	2	6M0J	2.45
*(SB14)+RBD	**1040**	**31**	**34**	**24**	**5**	7MFU_B	1.70
*(Sb14+Sb68)+RBD	**1663**	47	52	23	8	7MFU	1.70
Sb16+RBD	**1003**	26	29	8	1	7KGK	2.60
Sb45+RBD	**976**	27	33	15	4	7KGJ	2.10
*(Sb45)+RBD	**1010**	26	35	14	4	7KLW_B	2.60
*(Sb68)+RBD	**640**	21	17	9	4	7KLW_C	2.60
*(Sb45+Sb68)+RBD	**1650**	47	52	23	8	7KLW	2.60
*(VHH-E)+RBD	**821**	29	27	13	1	7KN5_C	1.87
*(VHH-U)+RBD	**625**	16	20	17	0	7KN5_E	1.87
*(VHH-E+VHH-U)+RBD	**1446**	45	47	30	1	7KN5	1.87
H11D4+RBD	**599**	20	20	11	4	6YZ5	1.80
H11H4+RBD	**637**	17	19	5	4	6ZBP	1.85
(CR3022)+RBD	**991**	19	20	7	4	6XC7_HL	2.88
VHH72+RBD	**796**	21	25	9	2	6WAQ_A	2.20
Nb20+RBD	**705**	22	21	9	4	7JVB	3.29
Nb6+Spike	**788**	24	21	9	1	7KKK_D	3.03
Sb23+Spike	**772**	21	22	5	0	7A25_D	3.06
Sb23+Spike	**585**	18	21	5	0	7A29_D	2.94
Ty1+Spike	**795**	23	26	3	0	6ZXN_D	2.93
C144+Spike	**689**	22	24	7	0	7K90_H	3.24
C002+Spike	**728**	22	23	7	1	7K8S_H	3.40
*(REGN10933)+RBD	**935**	28	30	9	1	6XDG_BD	3.90
*(REGN10987)+RBD	**607**	21	19	5	0	6XDG_AC	3.90
*(REGN10933+ REGN10987)+RBD	**1542**	49	49	5	0	6XDG	3.90

Extended Data Figure Legends.

Extended data Fig. 1. SEC profiles reveal direct interaction of sybodies with RBD. a, b, Sybodies and RBD were analyzed on either Shodex-KW-803, or **c, d, e, f** Shodex-KW-802 column. **a,** Sb14 and RBD, **b,** Sb15 and RBD, **c,** Sb16 and RBD, **d,** Sb45 and RBD, or **e,** Sb68 and RBD were mixed in equal concentrations (50 µg in 100 µl), incubated at 4 °C overnight, and then analyzed.

Extended Data Fig. 2 | Electron density maps of sybody CDR match models. Electron density maps (2mFo-DFc) (blue) for CDR loops and those residues in contact with RBD are superposed on models. **a**, Sb16 on RBD surface, Resolution=2.6 Å, Rfree=0.277. **b,** Sb45 on RBD surface, Resolution=2.3 Å, Rfree=0.216. **c,** Sb68 on RBD surface, Resolution=2.6 Å, Rfree=0.255. **d,** Sb14 on RBD surface, Resolution=1.7 Å, Rfree=0.215. **e,** Sb16 alone, Resolution=2.1 Å, Rfree=0.259. Maps are contour at 1.0 σ, CDR1 loop as pink, CDR2 loop as orange, CDR3 loop as red, non-CDR residues as lime, and RBD is in background as gray.

Extended Data Fig. 3 | Comparison of liganded vs unliganded Sb16. A superimposition Sb16-alone ("unliganded", free, green) and Sb16+RBD ("liganded", complexed, slate) reveals the large movement of CDR2 loop (about 6 Å). specifically, Y54 moved about 15 angstroms and dipped into a binding pocket which is surrounded by epitopic residues Q409, E406, D405, R403, G416, K417, I418, N422, L455, Y453, Y495. (RBD surface is gray).

Extended Data Fig. 4 | CryoEM data processing and classification of SB45+S-6P. **a,** Negative stain image of Sb45 + S-6P. **b,** 2D classes of averages. **C,** 3D classification (Ab-initio reconstruction) to recognize two forms of S-6P. **d,** After homogeneity refinement and final non-uniform refinement, Gold-standard Fourier shell correlation (FSC) presents the resolution (at 0.143) for the map of 1-up, 2-down form of S-6P. **e,** For the map of 2-up, 1-down form of S-6P. **f,** Local resolution estimation for 1-up, 2-

down form of S-6P. **g,** Local resolution estimation for 2-up, 1-down form of S-6P, color as the resolution scale bar.

Extended Data Fig. 5 | Cryo-EM electron density map of Sb45 on S-6P. Extracted electron density of Sb45 on S-6P from cryo-EM map. **a,** Sb45-X on RBD-A (up) of S-6P (1-up, 2-down). **b,** Sb45-Y on RBD-B (down) of S-6P (1-up, 2-down). **c,** Sb45-Z on RBD-C (down) of S-6P (1-up, 2-down). **d,** Sb45-X on RBD-A (up) of S-6P (2-up, 1-down). **e,** Sb45-Z on RBD-C (down) of S-6P (1-up, 2-down). Electron densities (blue) are contoured at 1.5 **σ**. Sb45-X, Sb45-Y and Sb45-Z are colored red, orange, and firebrick respectively; RBD-A, RBD-B, and RBD-C are colored pale green, pale cyan, and light pink respectively. RBDs are shown in surface as gray. The local resolution for Sb45 is in a range of 4.5-7.0 Å and CC (Correlation Coefficient) are lower, the side chains of CDR3 loops are seen in good density, tightly binding to RBD.

Extended Data Fig. 6 | Superposition of X-ray models on spike maps. All spike models are based on 7N0G/7N0H (Sb45+S-6P). **a,** Superposition of Sb16-RBD (7KGJ, slate) on the four forms of spike. **b,** Binding of both Sb16 and Sb68 on the 2-up and 3-up forms of spike. **c,** Superposition of Sb45-RBD (7KGK, cyan) on the four forms of spike. **d,** Binding of (Sb45 and Sb68)-RBD (7KLW) on 2-up and 3-up forms of spike. **e,** Sb14-RBD (7MFU, marine blue) can only access to the three "up" forms of spike. **f,** Sb68 (blue purple) can only access the 2-up and 3-up forms. **g,** Superposition of (Sb14 and Sb68)-RBD (7MFU) on 2-up and 3-up forms of spike. A-, B- and C-chains of spike are colored as white, grey-80%, and grey-60% respectively.

Extended Data Fig. 7 | Antibody classifications based on site of RBD interaction. a, Definition of Classes [1]. Representative Fab (only showing the variable domains), nanobody, or sybody illustrated with different colors show the classifications. RBD is in gray and two N-glycans (N165 and N343 in red). Sb14

(marine blue) belongs to Class-1, Sb16 (slate blue), and Sb45 (cyan) belong to Class-2, and Sb68 (purple blue) falls in Class-4 overlapping with VHH72, VHH-E, and CR3022. **b,** Comparison of the ternary sybody structures (Sb45+RBD+Sb68, 7KLW) and (Sb14+RBD+Sb68, 7MLU) with the ternary nanobody structure (VHH-E+RBD+VHH-U, 7KN5), (Koenig, *et al.* *Science*, 2021-01-12). Sb45 in cyan, Sb68 in purple blue, RBD in gray, VHH-E in green, VHH-U in blue. CDR1, CDR2, and CDR3 of Sb45, CDR3 of Sb68 are highlight as pink, orange, and red. Three views of 90 degrees of rotation. CDR3 and CDR2 loops of Sb45 interacts with both sides of the RBD, while VHH-E uses only the extended CDR3 loop on the side. Sb68 binds lower than VHH-U while VHH-E is closer to VHH-72. The total BSA ($Å^2$) for 7KLW, 7MFU and 7KN5 are 1,650, 1,663, and 1,446 respectively. **c,** The interactions between Sb14 and Sb68 in the ternary structure (Sb14+RBD+Sb68, 7MFU): Y57-E44, G55-E44, and T54-H108.

Extended data Fig. 8 | Purity of sybodies, RBD, and S. a, b, c, d, e, Monomeric sybodies as indicated were purified on SRT-10C-SEC100 columns. Elution time of each sybody is indicated above each peak. The *y* axis represents A_{280} nm absorbance units (mAU). **f,** SEC profile of trimeric spike protein (Superose^{TM} 6 10/300 GL). **g,** SDS-PAGE image of purified sybodies, RBD and S.

Extended data Fig. 9 | Sybodies, RBD, and spike protein reveal unique thermal stability. T_m of each of the indicated purified proteins was determined by thermal melt analysis as described in Methods. Note the biphasic behavior of the trimeric S protein.

Supplementary Reference

1. Barnes, C.O. et al. SARS-CoV-2 neutralizing antibody structures inform therapeutic strategies. *Nature* (2020).

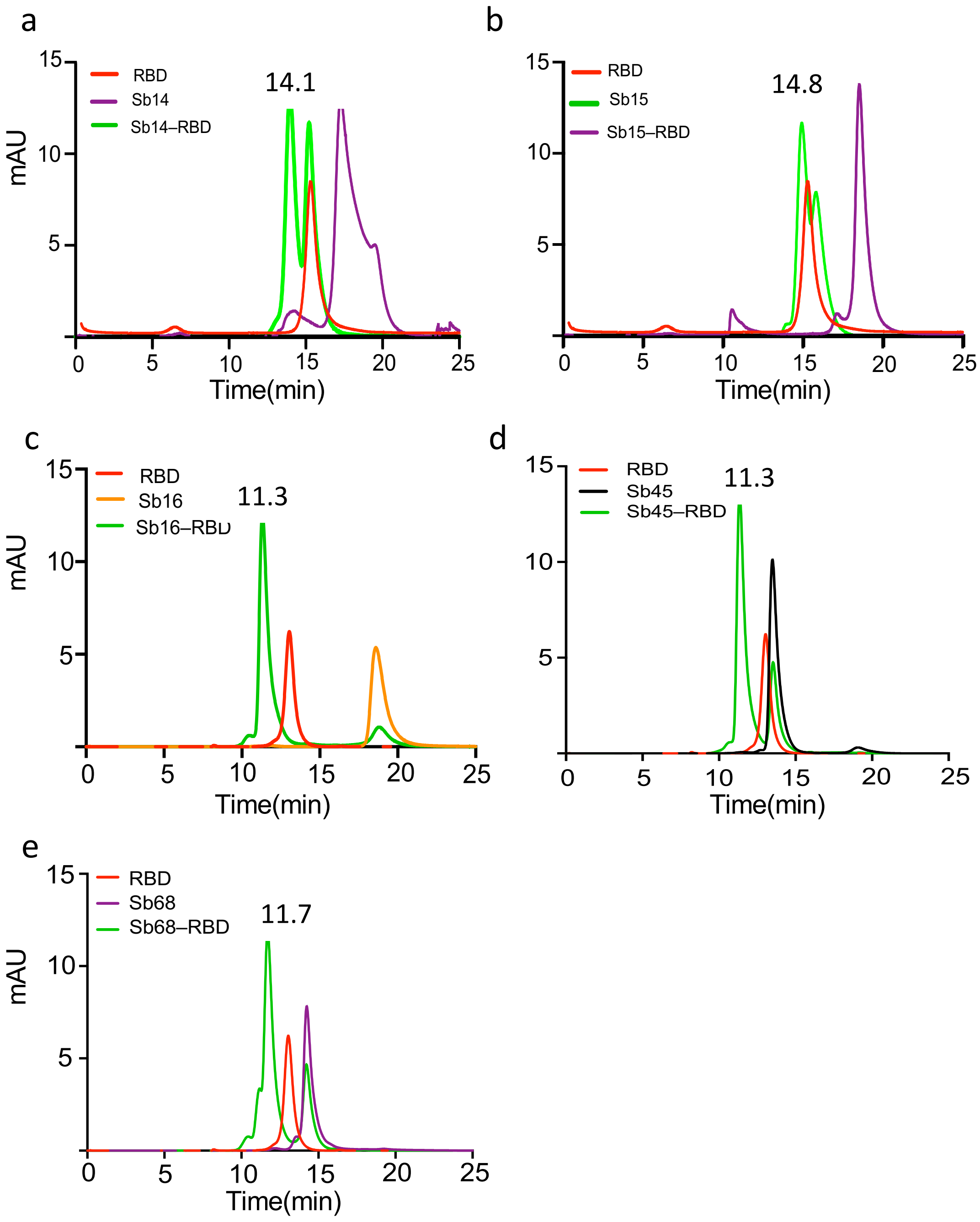

Extended Data Fig. 1

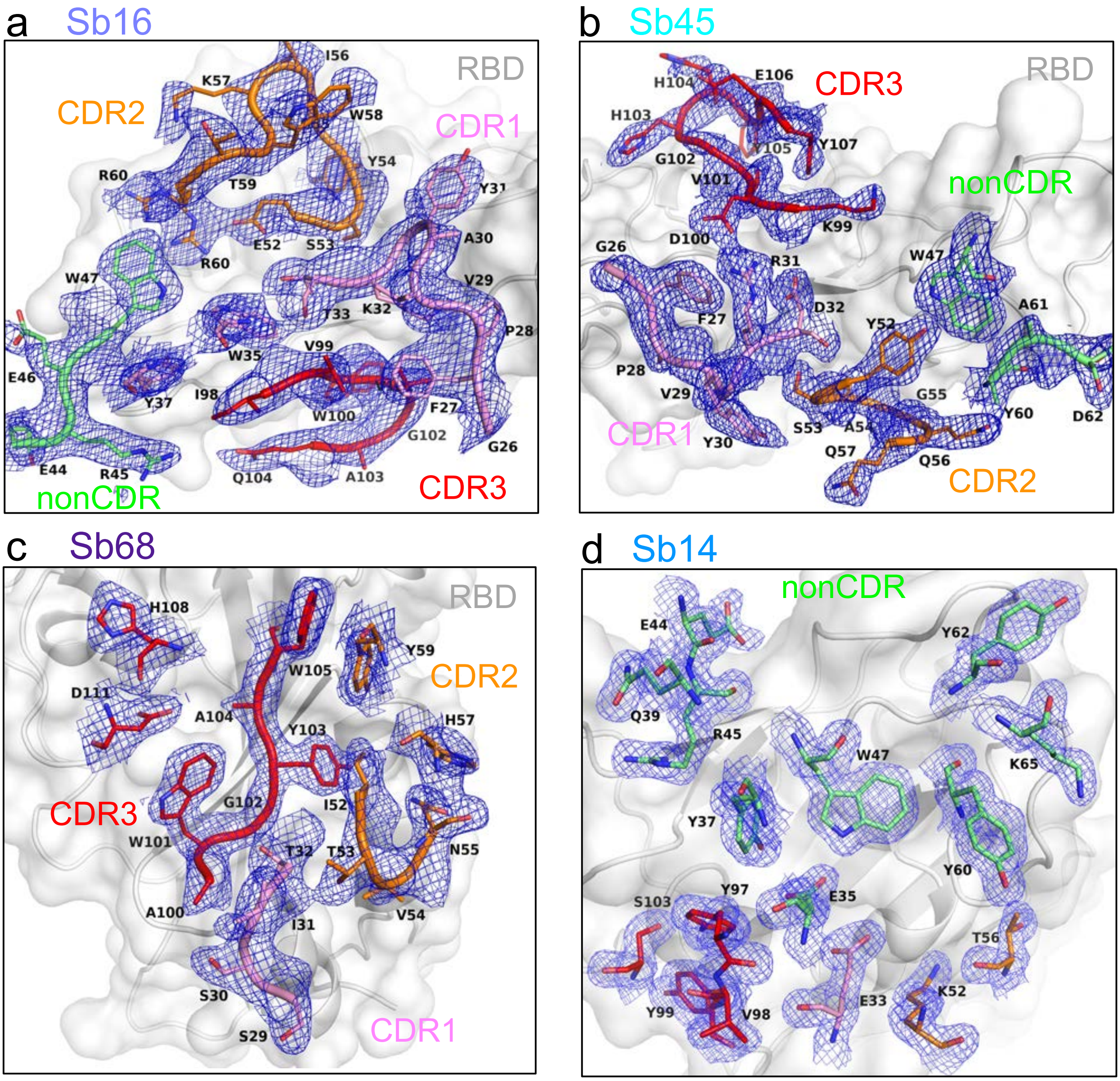

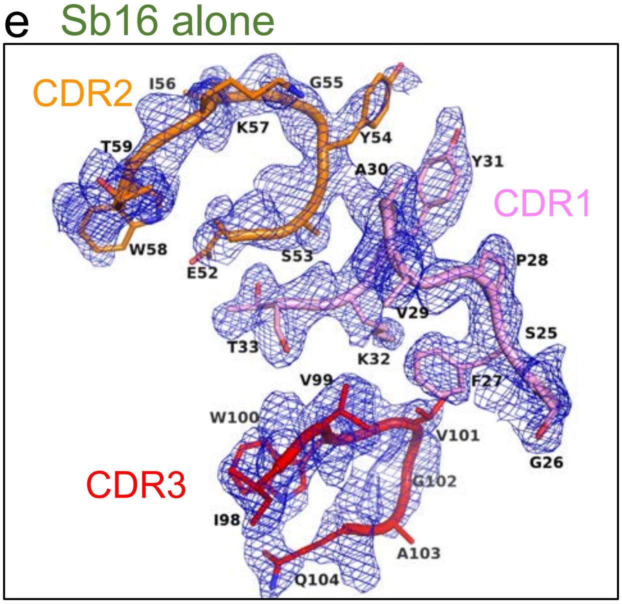

Extended Data Fig. 2.

a

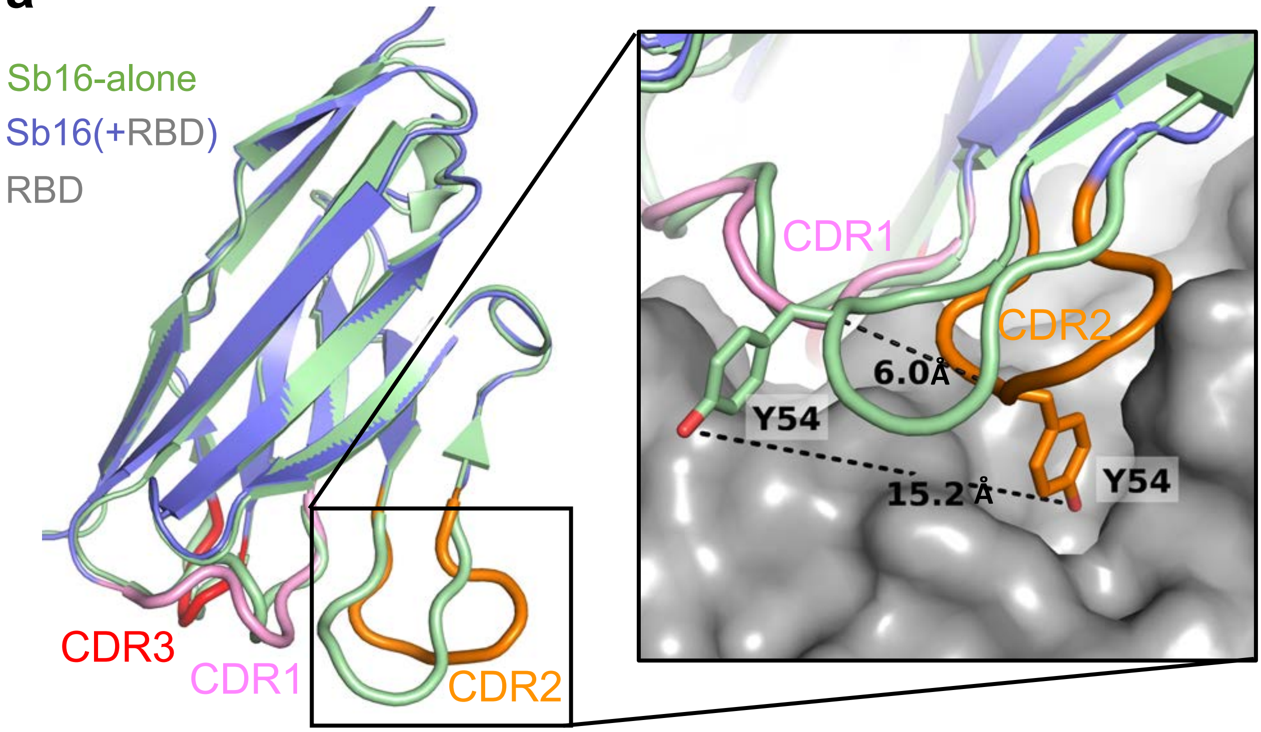

Extended Data Fig. 3

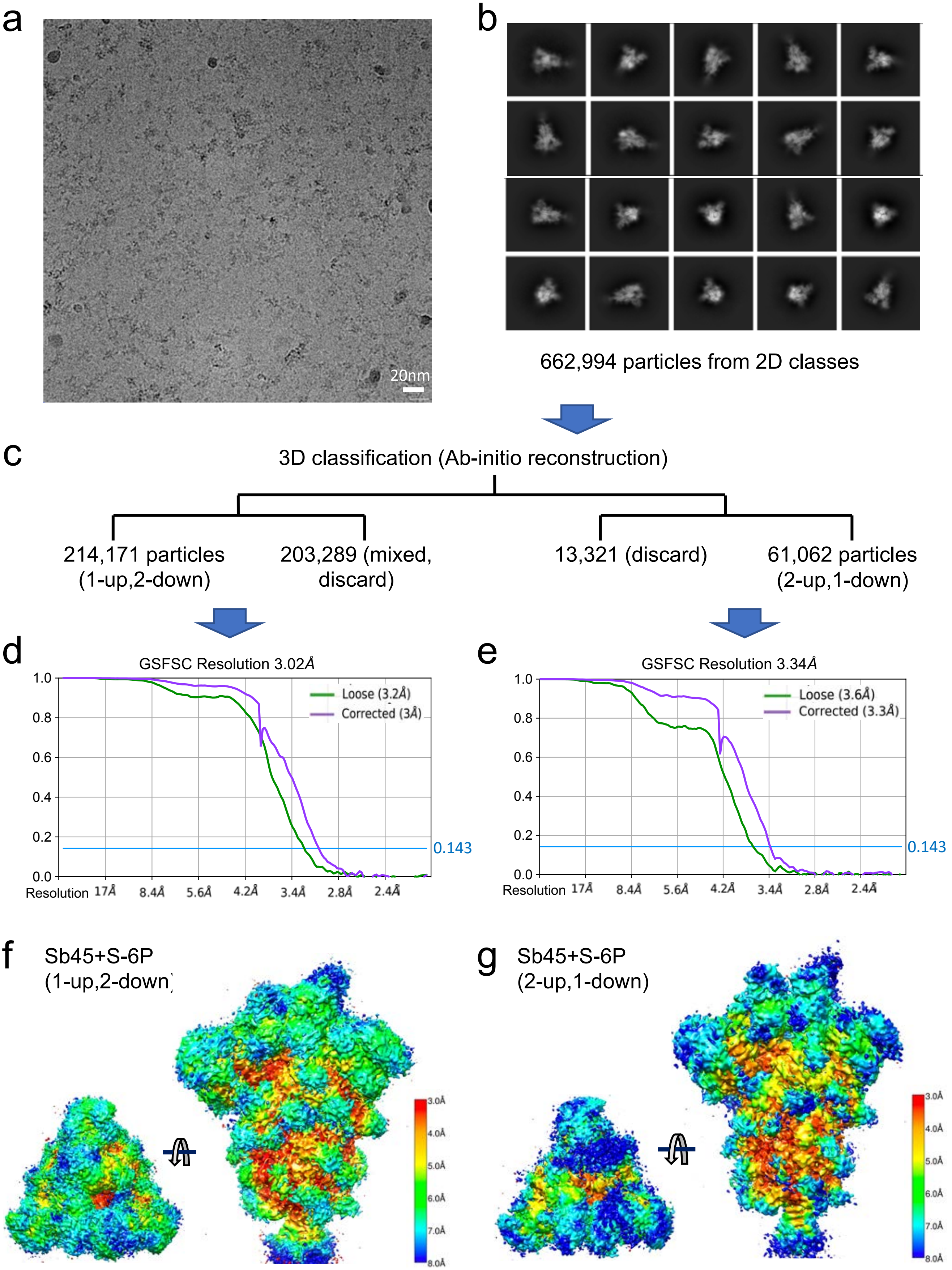

Extended Data Fig. 4.

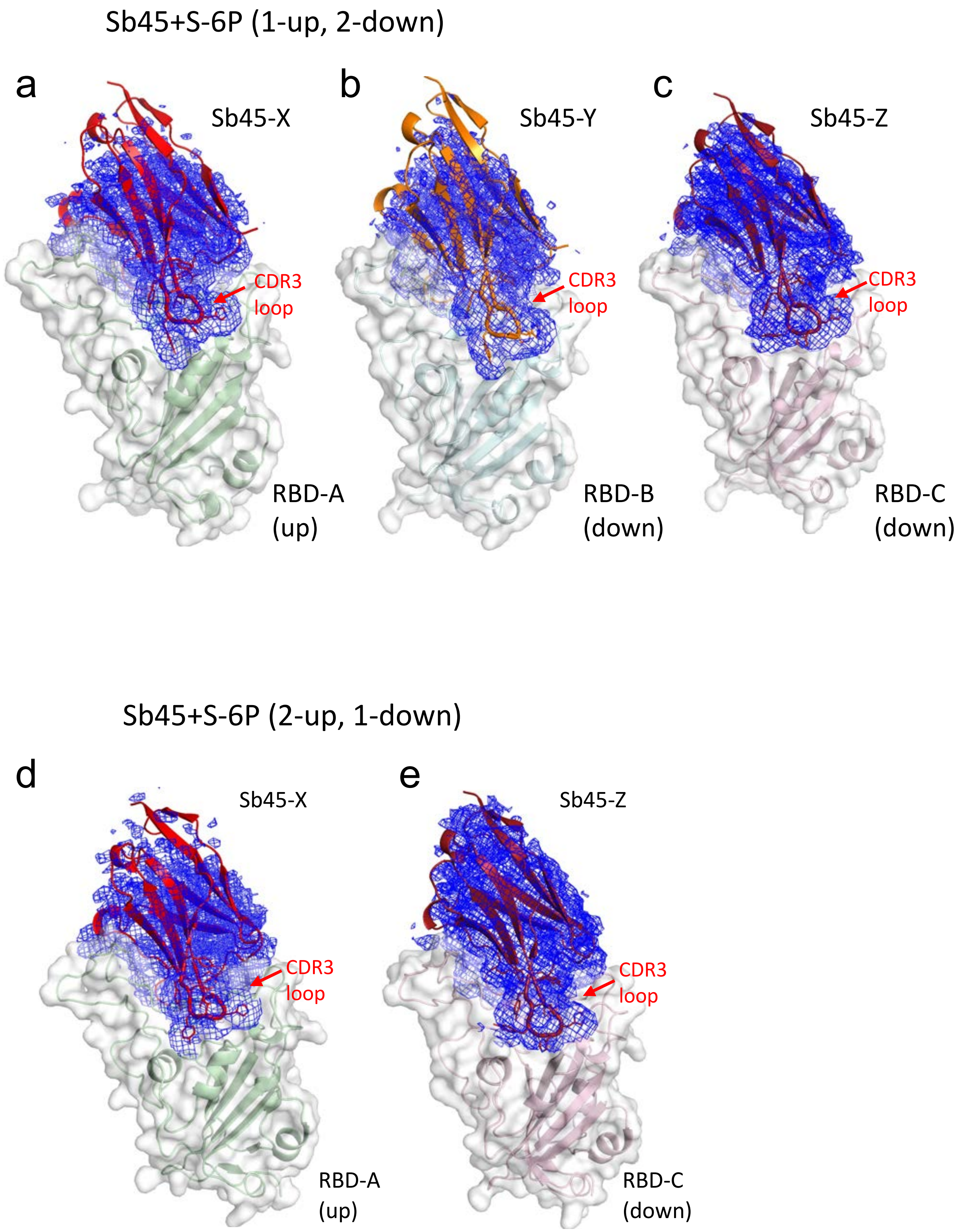

Extended Data Fig. 5

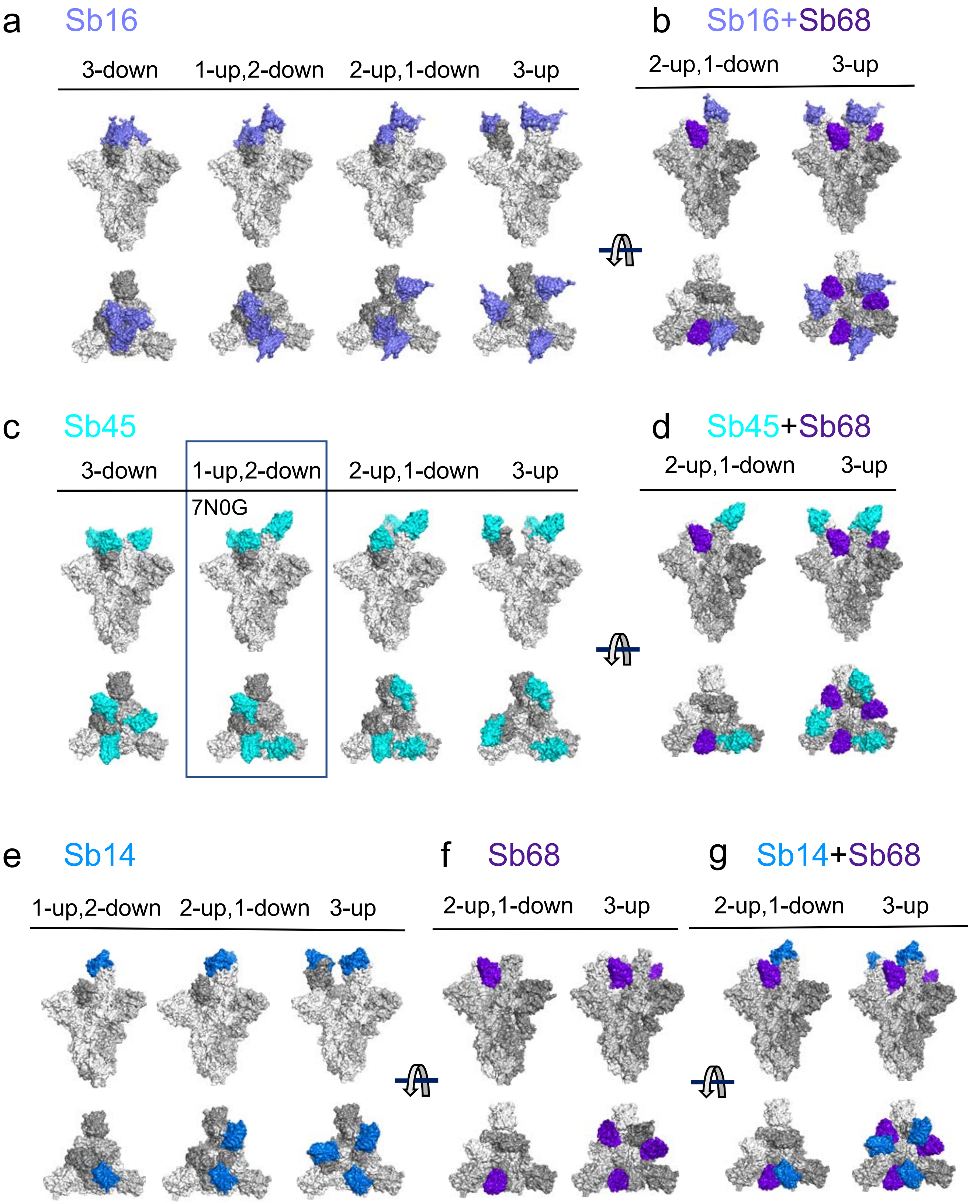

Extended Data Fig. 6

a

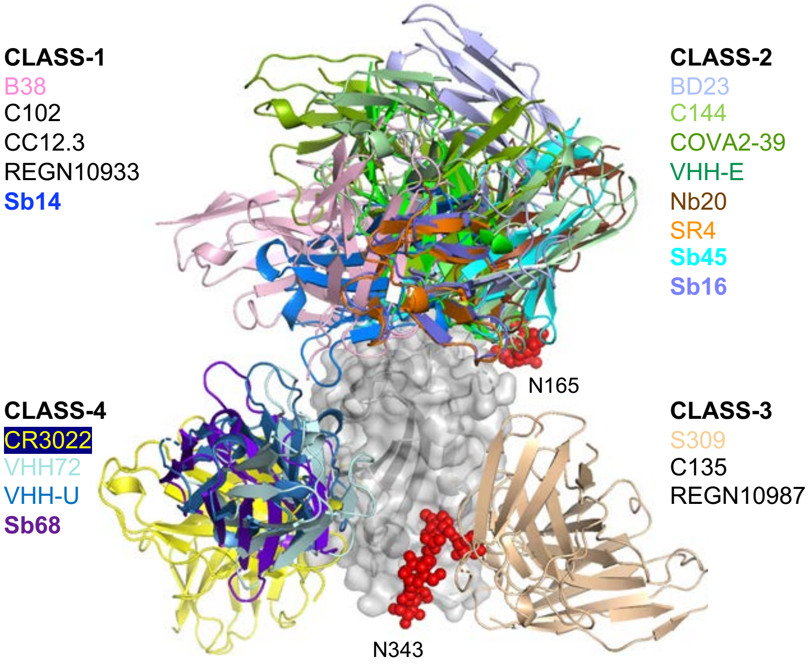

Barnes	Definition
CLASS-1	Blocks ACE2 and binds “up” RBD
CLASS-2	Blocks ACE2 and binds “up” and “down” RBD
CLASS-3	Does not block ACE2 but binds “up” & “down” RBD
CLASS-4	Does not block ACE2 but binds “up” RBD only

b **7KLW and 7MFU vs 7KN5**

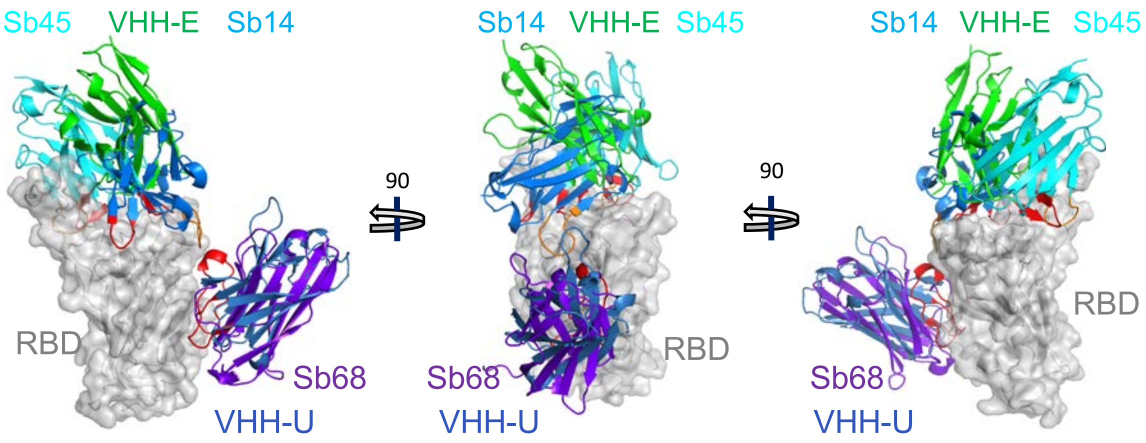

c **7MLU**

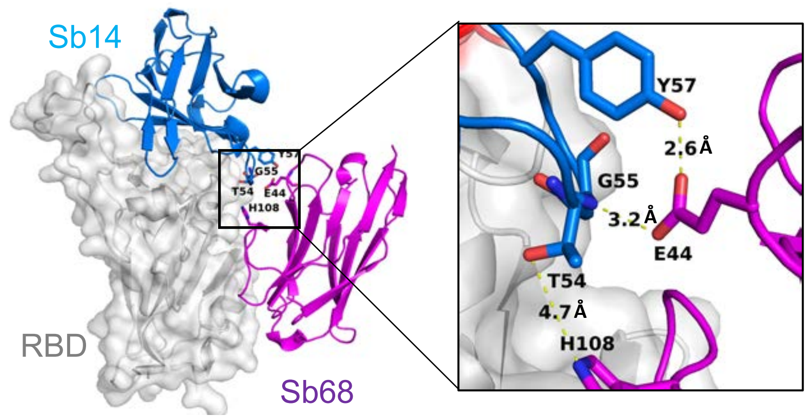

Extended Data Fig.7

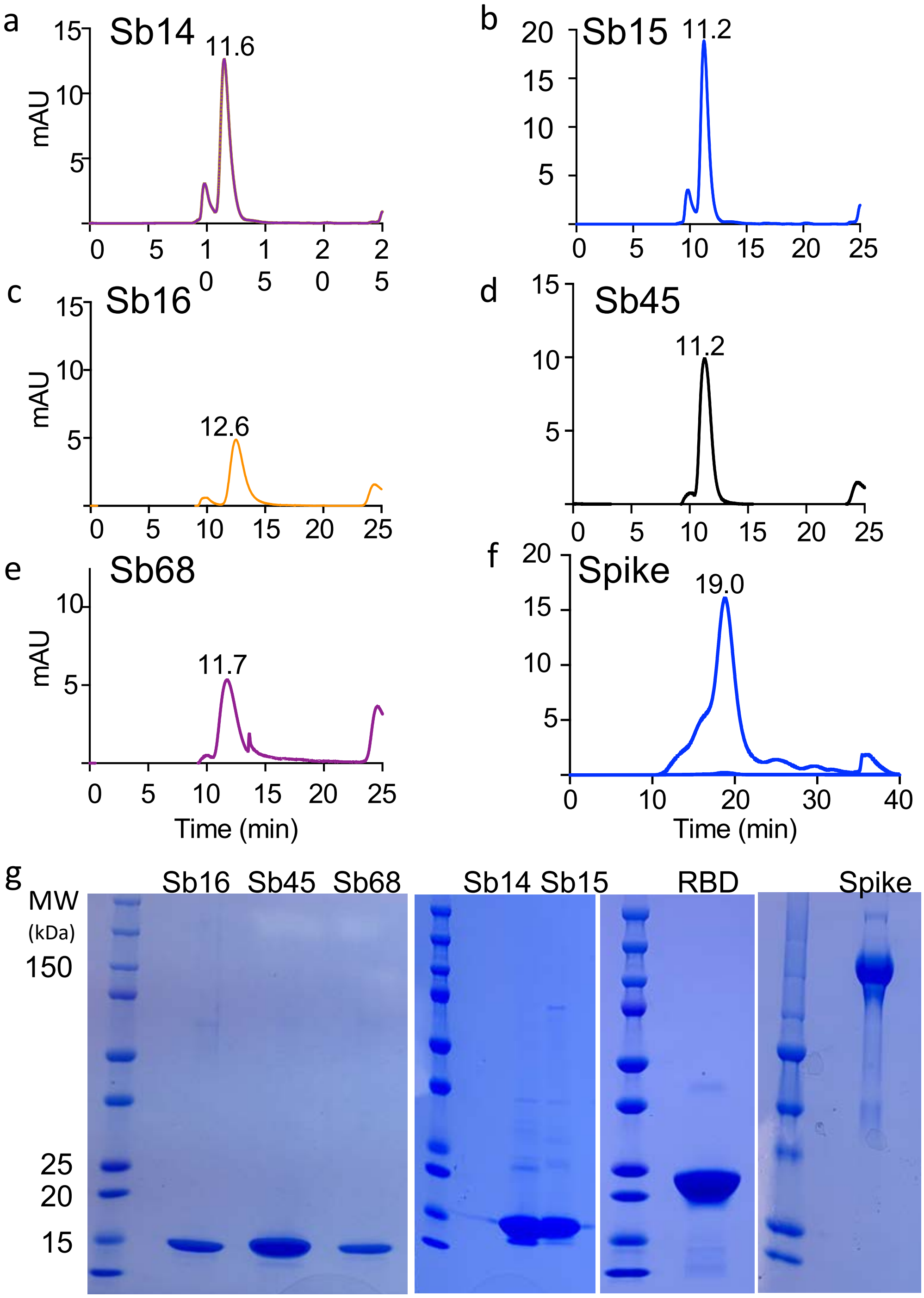

Extended Data Fig. 8

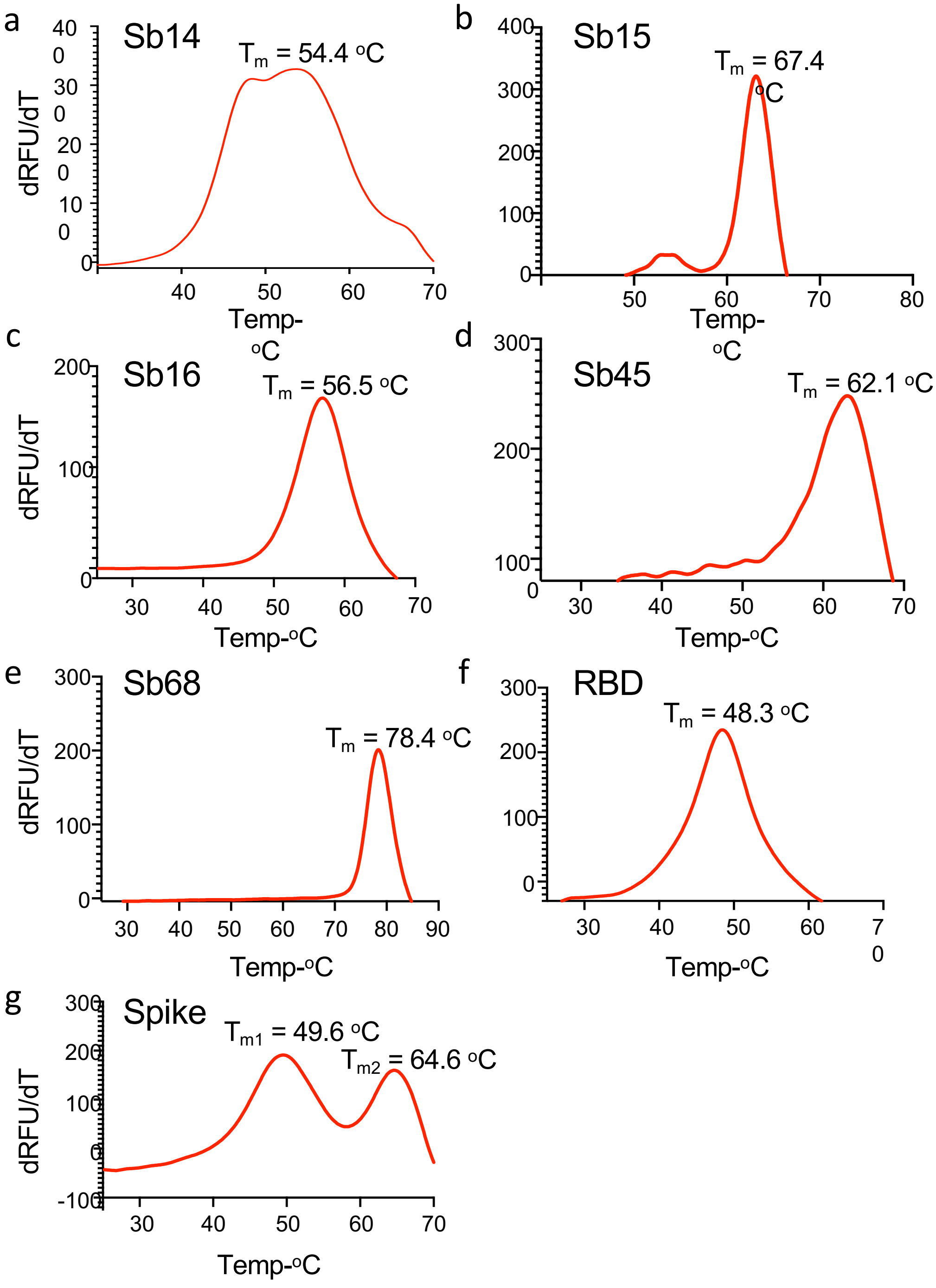

Extended Data Fig. 9
